# Selective insensitivity to income held by the richest

**DOI:** 10.1093/pnasnexus/pgae333

**Published:** 2024-09-17

**Authors:** Barnabas Szaszi, Hooman Habibnia, Josephine Tan, Oliver P Hauser, Jon M Jachimowicz

**Affiliations:** Institute of Psychology, ELTE, Eötvös Loránd University, Budapest, 46 Izabella street, 1064 Budapest, Hungary; Institute for Cognition and Behavior, WU Vienna University of Economics and Business, 1 Welthandelsplatz, 1020 Vienna, Austria; Stanford Graduate School of Business, Stanford University, 655 Knight Way, Stanford, CA 94305, USA; Department of Economics and Institute for Data Science and Artificial Intelligence, University of Exeter, Streatham Court, Rennes Drive, EX4 4PU, United Kingdom; Organizational Behavior Unit, Harvard Business School, Harvard University, Boston, MA 02241-2275, USA

**Keywords:** economic inequality, perception, misperception, redistribution

## Abstract

The misperception of income inequality is often touted as a critical barrier to more widespread support of redistributive policies. Here, we examine to what extent and why (mis)perceptions vary systematically across the income distribution. Drawing on data from four studies (*N* = 2,744)—including a representative sample and preregistered incentive-compatible experiments—we offer converging evidence that people specifically underestimate the amount of income held by the top of the income distribution. While this selective underestimation is likely driven by multiple mechanisms, including systemic factors, we find that cognitive biases contribute to the observed pattern of results. The rise of inequality in many developed countries has been documented before, and the fact that this growing inequality is largely driven by the outsized gains of the richest individuals may pose new challenges previously underappreciated: our theory and findings highlight that cognitive biases pose a key obstacle to people's recognition of the concentration of income among the richest individuals, and may potentially distort their preferences for redistribution. We conclude by discussing future directions for research and the importance of incorporating behavioral and cognitive limitations into the design of redistributive public policy.

**Significance Statement**

Across four studies, we show that people uniquely underestimate incomes concentrated at the upper end of the income distribution, leading to a systematic underestimation of inequality. With inequality's rise being largely driven by the outsized gains of the richest, this selective underestimation may contribute to people's lower support for redistribution. We find that this underestimation is partially driven by cognitive biases, suggesting correcting people's perceptions about the income of the richest may be challenging. Finally, our findings indicate that inequality should not be treated equally across the income distribution. Beyond summary measures of inequality such as the Gini coefficient, research and policy may need to shift toward being more sensitive to where inequality is concentrated.

## Introduction

While within-country economic inequality has risen around many parts of the world in recent decades, its growth in many developed countries is often driven by those at the very top of the income distribution (1–3). For instance, in the United States, between 1980 and 2020, the share of incomes held by the top 1% nearly doubled from 10 to 19%, while the top 10%'s income share—excluding the top 1%—increased far more modestly, from 24 to 27% (3). Critically, as inequality rose, the fraction of people supporting redistribution in the United States showed no significant increase since the 1970s (4–7).

Prior research suggests that to redress inequality and garner support for redistribution, people may need to recognize the existence and degree of inequality, particularly its growth at the very top. However, the perception of inequality often does not match reality (8–13), with some studies finding evidence for underestimation (e.g. 8, 14, 15), while others show support for overestimation of inequality (e.g. 11, 16). In the current research, we focus on the top of the income distribution—given their outsized gains over the last few decades—and examine whether (and why) the perceptions of the incomes of this group in particular are commonly misperceived, and discuss the policy consequences of our findings.

Theories across the social sciences make competing predictions about the perception of incomes at the very top of the income distribution. For instance, the *upward social comparison hypothesis* (17) suggests that people are more prone to making upward than downward social comparisons. That is, people are hypothesized to be more likely to pay attention to information about individuals richer than them than poorer than them, which may lead to greater accuracy for estimates of the top of the income distribution. Similarly, the richest individuals—being the most successful in economic terms—are often overrepresented in the media. As the media influences peoples’ perceptions more broadly (18), such overrepresentation can provide an important source of information in people's judgments of inequality and perhaps contribute to a more accurate perception of the resources held by the richest (10).

In contrast, other theories predict lower overall accuracy at the upper end of the income distribution. The *network hypothesis* suggests that because people are often geographically segregated by income and wealth, many of them rarely interact with those from other social classes, particularly with individuals from the top of the income distribution (19–21). Because people's perceptions of incomes are shaped by personal experience (22) including their ideology (23), this perspective suggests they may underestimate the incomes of the richest individuals. In addition to geographical segregation, theories on cognitive biases also shed light on why people commonly underestimate the incomes of the very top of the income distribution (see also 24, 25). Scope insensitivity refers to people's tendency to be insensitive to the quantity of objects, such that an additional unit is perceived and valued less than the previous one (26). Because scope insensitivity has been documented across several domains—including visual and auditory perception, social psychology, and economics (27–30)—it may also occur in the domain of income estimations.

In sum, there are several potential mechanisms—both systemic and cognitive—that may shape how people perceive incomes by the upper echelons of the income distribution, but it is unclear how or when this occurs. Consequently, in the present research, we examine whether people over- or underestimate incomes at the upper end of the income distribution, and whether this effect is unique to this part of the distribution. In addition, we examine potential underlying mechanisms of the observed pattern of results. To do so, we combined several complementary methods and data. First, we conducted a preregistered cross-sectional study, as well as a replication with an incentivized US sample, which found that people strongly and consistently underestimate the resources of the top 1%—but this underestimation does not equally extend to other, lower income percentiles. Next, we conducted two preregistered and incentive-compatible experiments where we systematically manipulated income distributions. In these studies, we replicate our findings—i.e. participants were largely insensitive to changes in incomes of the top 1%—and show that this effect is unique to the top of the income distribution. Additional data from these experiments further suggest that cognitive processes in part contribute to the observed findings; more precisely, while our data are largely consistent with the predictions of scope insensitivity, it is likely that several factors—including both cognitive and systemic elements—influence people's judgments about inequality pertaining to the richest individuals in particular.

## Results

### Study 1a: cross-sectional study

**Fig. 1.**
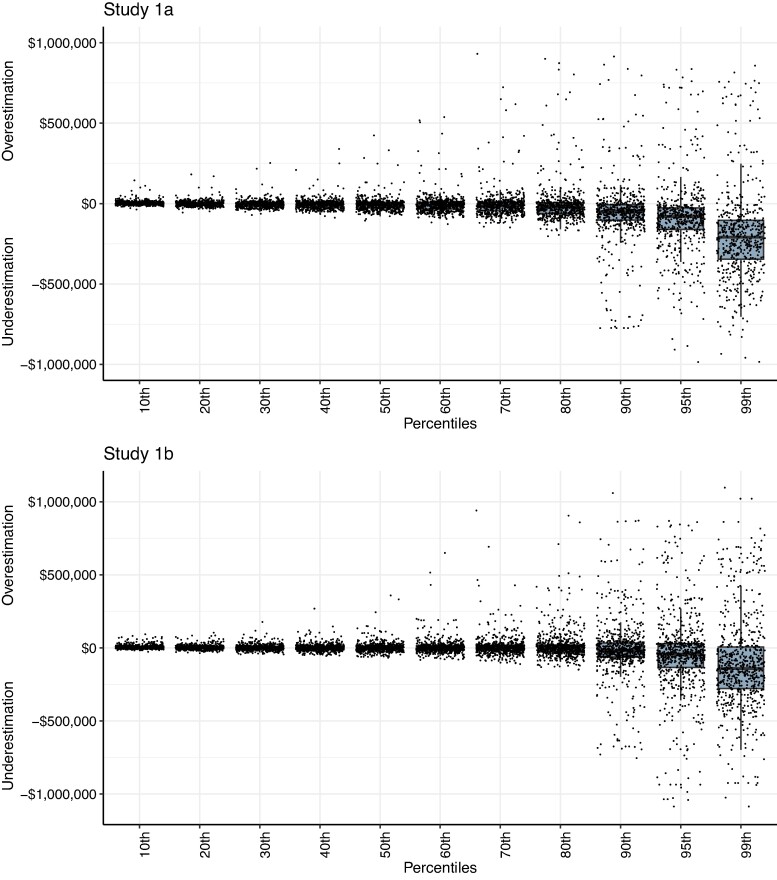
The difference between participant estimates of income average per quintile and the actual income average per quintile in Study 1a (top panel) and Study 1b (bottom panel). Note that data for Figure 2 are specific to responses from only the *top 1% treatment* condition. As this figure shows, participants underestimated incomes held by the top 1%, but were relatively accurate—and if anything, somewhat overestimated—incomes held by the bottom 80% of this fictional society.

In Study 1a, we aimed to test whether people under- or overestimate the amount of income held by the top of the income distribution and explore whether this misperception is unique to this group. 990 US residents (52.9% female, *M*_age_ = 44.27 years) recruited from luc.id completed the study online. Those who successfully completed the attention check were provided with an explanation of income percentiles. Next, participants were asked to self-report the US county they currently live in and then estimated the minimum annual household income thresholds of the 10th, 20th, 30th, 40th, 50th, 60th, 70th, 80th, 90th, 95th, and 99th percentiles of this county.^a^ We subsequently calculated how much perceived income thresholds differed from the objective income thresholds in each county, and in each percentile, which we calculated using data provided by the Economic Policy Institute (31, 32). Using Wilcoxon’s tests clustered by participants, we found that participants underestimated the income thresholds of the top 1% (*P* < 0.001, *Z* = 12.490, *r* = 0.513), but this underestimation did not equally extend to lower percentiles (see Figure 1, top panel; see also Table S1). For example, people underestimated the income threshold of the top 1% more so than that of the top 5% (*P* < 0.001, *Z* = 13.465, *r* = 0.553). Detailed results and robustness checks for this and all subsequent studies are reported in the Supplementary Materials.

### Study 1b: preregistered replication in an incentivized US sample

In Study 1b, we conducted a preregistered replication of Study 1a and financially incentivized accurate responding. The preregistration is available at https://aspredicted.org/RPG_SC7. We obtained a sample aimed to be representative by gender and race, as filtered by Prolific. 834 US citizens (*M*_age_ = 37.83, 49.1% female) completed the study online. The procedure of the study was identical to Study 1a with one key difference. Participants were told that they would earn an additional bonus payment of $0.50 if they completed one version of the so-called Bourdon test successfully, a task in which participants were asked to count the amount of “2”s present in a large table consisting of numbers. Participants who provided relatively accurate estimates (±5 from the real value) earned the bonus payment and could continue with the survey. Afterward, participants were informed that they could use this earned money to bet on their future performance in estimating income distributions, and if they were within the top 25% of all participants regarding the accuracy of the estimations, they would earn four times the amount of money wagered. 74% of the participants wagered some money (median amount wagered = $0.25).

Using identical means to Study 1a, we calculated how much perceived income thresholds differed from objective income thresholds in each percentile. Our results showed the same pattern of results as before: participants underestimated the income thresholds of the top 1% (*P* < 0.001, *Z* = 7.520, *r* = 0.282), but this underestimation was selective, i.e. it did not equally extend to lower income percentiles (see Figure 1, bottom panel; see also Table S3). Note that these results are identical when looking only at participants who wagered all their bonus money (24.33%) as well as participants who did not wager money at all (25.60%). These results suggest that participants were unable to provide more accurate guesses simply by trying harder. In additional supplementary analyses, we also find that these effects are not significantly moderated by political orientation.

Taken together, in Studies 1a and 1b, we found robust evidence across two cross-sectional studies from the United States that people systematically underestimate income of the top 1% and that this effect dissipates as we descend the income distribution. To further evidence the robustness of our findings, we aimed to replicate these results in Studies 2a and 2b using experimental methods. This approach also allows us to examine whether and how cognitive biases contribute to this misperception, beyond potential systemic factors.

### Studies 2a and 2b: preregistered lab experiments with incentivized US sample

In Studies 2a and 2b, we developed an experimental design that allowed us to systematically manipulate the shape of the income distribution. After responding to the consent form, participants were asked to review the incomes of 100 individuals from a fictional society. Participants were required to click on the picture of each individual in any order of their choice, after which the income of the given individual appeared on the screen for 1.5 seconds. After finishing this task, participants were asked to assess the average income for each quintile of the income distribution of this fictional society and were offered an additional $0.50 bonus if their answers were within the top 25% accuracy among all participants. Participants subsequently repeated the same procedure for another fictional society.

We used a mixed within–between-participants design, where each participant saw two fictional societies in random order, one representing a *control* condition and the other representing a *treatment* condition (in Study 2a, only the *top 1% treatment*; in Study 2b, either the *top 1% treatment* or *top 10% treatment*). In the *control* condition, the distribution of the presented 100 incomes was broadly representative of the United States with respect to the mean and the percentage of people belonging to different income groups. The exact distributions are available in the Supplementary Materials. In the *top 1% treatment condition*, we used the same distribution as in the *control* group up until the richest individual, whose income was significantly increased. That is, the incomes of 99 individuals were the same between the *control condition* and the *top 1% condition*, and the only difference was the income of the richest person. In the *top 10% condition*, we used the same distribution as in the control group for the bottom 90 individuals and for the richest individual, but significantly increased the income of the richest 2–10% of individuals. That is, the incomes of 91 individuals were the same between the *control* and the *top 10% condition*, and the only difference was the increased income of the richest 2–10% of individuals. After participants viewed each fictional society, they were asked to estimate their perception of the income distribution—consistent with Eriksson and Simpson (33)—separately for each quintile, as follows: *What do you think is the average income of the richest/second/middle/fourth/poorest 20% of the individuals in the presented society?* Note that in asking about income *quintiles*, we also intended to reduce the likelihood that experimenter demand effects would drive our results (34).

### Study 2a

The aim of Study 2a was to test whether individuals were sensitive to variations in the incomes held by the top 1%. Accordingly, each participant in the study viewed two fictional societies in random order, one representing the *control* group and the other the *top 1% treatment* condition. In the *top 1% treatment condition*, we randomly varied between-participants the income of the richest earner to be either 35, 40, 45, or 50% of total income. We recruited participants via Prolific, and a total of 455 passed the attention check and completed the study (48.6% female, *M*_age_ = 36.44 years). The preregistration of this study is available at https://aspredicted.org/MYF_KNM.

**Fig. 2.**
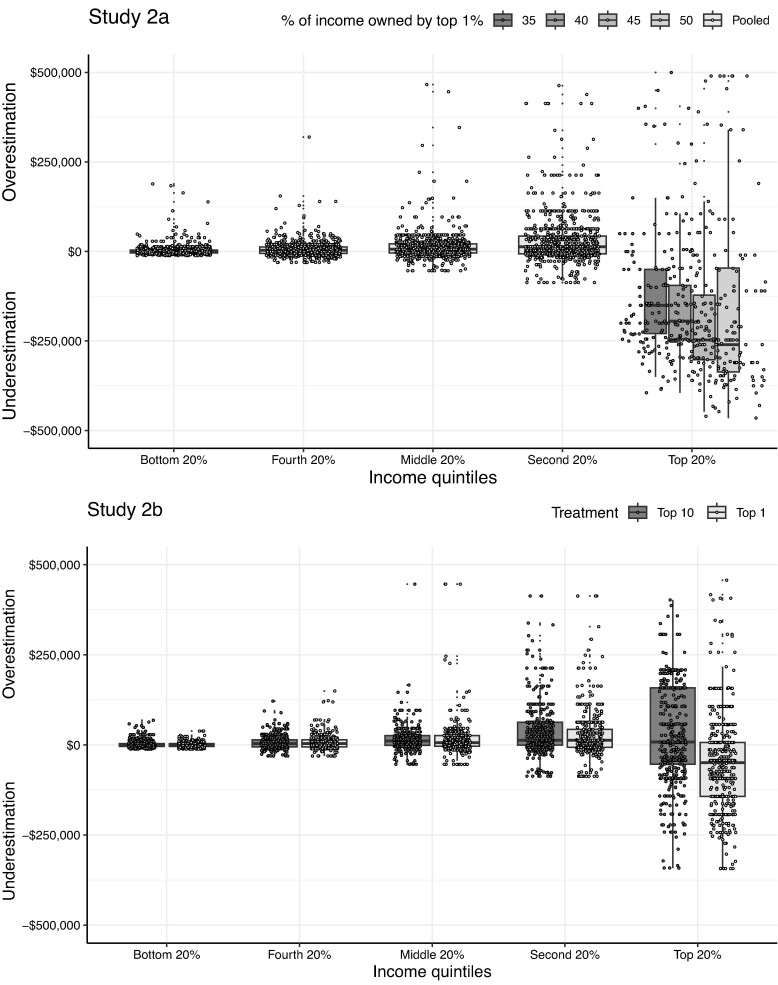
(In)accuracy of participant's estimations of income thresholds by income percentile. Top panel reflects data from Study 2a, and bottom panel reflects data from Study 2b. Across both studies, we find that participants underestimated the income thresholds of the top 1% more so than all other percentiles.

First, we compared participants’ perceptions of each income quintile to objective data. To do so, we collapsed data from participants’ responses in both fictional societies (*control* and *top 1% treatment* groups) and used a clustered Wilcoxon signed-rank test clustered by participants to compare the differences in participants’ perceptions of each income quintile with objective data for each income quintile. In line with our prior studies, we find that participants underestimated the average income of the top 20% (*P* < 0.001, *Z* = 7.870, *r* = 0.297), while they did not underestimate—and in fact, often overestimated—incomes of all other, lower quintiles (see the top panel of Figure 2 and Table S18).

Second, we compared participants’ estimated difference between the *control* and *top 1% treatment* conditions to the objective difference between the conditions with respect to the top quintile income. Our analyses revealed that the perceived difference (*Mdn* = 50,000, *P* < 0.001, *Z* = −8.212, *r* = 0.385) in top quintile incomes between the *control* and *top 1% treatment* conditions was significantly smaller than the objective difference (*Mdn* = 253,965). That is, participants somewhat but insufficiently incorporated the income change of the top 1%.

Finally, we tested *how* sensitive participants were to changes to the income of the richest individual in the *top 1% treatment* condition. Recall that the incomes of the richest person in the top 1% treatment varied between-person, ranging from 35, 40, 45, or 50% of total income. In comparing perceptions of average incomes of the richest quintile, we can test how sensitive participants are to different income levels of the richest person in the fictional society. We tested whether participants’ estimate of the richest quintile varied as a function of how rich the richest person was, and did not find a statistically significant effect (*F*(3,451) = 1.084, *P* = 0.355; see also comparisons within top 20% quintile in the top panel of Figure 2).

Critically, it is unlikely that this effect is entirely driven by inattention to the income of the top 1% alone. Consider that participants were required to click on all individuals’ faces and observe their income one by one. In addition, we observe *some* difference in participants’ estimates of top income quintiles; when comparing the lowest (35% of total income) and highest (50% of total income) amounts of incomes held by the richest person in this fictional society, we find a small difference in perceptions (of 0.04%) which stands in contrast to the objective difference (of 8%). The detailed results of these additional analyses are reported in the Supplementary Materials.

In sum, in Study 2a, we replicated our earlier findings, showing that participants selectively underestimate at the top of the income distribution, and are not sufficiently sensitive to changes in the income of the top 1%. This study also provided evidence that this underestimation can occur even in the absence of systemic factors, suggesting that cognitive processes may also play a role—a possibility we investigate in more detail next.

### Study 2b

In Study 2b, we aimed to accomplish three objectives: first, to replicate the findings from Study 2a; second, to provide evidence in an experimental setting that the effect does not equally extend to lower echelons of the income distribution (i.e. the underestimations is smaller for the top 10% than for the top 1%); and third, to test whether—in line with predictions from scope insensitivity theory—participants’ affective response does not proportionally vary with differences in the amount of income.

To accomplish these aims, we made two changes to Study 2a. First, in addition to the *control* condition, participants were randomly assigned to one of two treatment conditions, either the *top 1% treatment* condition where the income of the richest individual was increased to have 35% of the total income, or the *top 10% treatment* condition where the incomes of the top 2–10% of richest individuals were increased to altogether have 35% of the total income. The rank order of the individuals remained the same even after the increase of the incomes of the top 2–10%. Second, at the end of the study, we adapted measures from the scope insensitivity literature to capture participants’ affective responses to varying income levels (28). Each participant was shown 12 quasi-randomly generated incomes in random order, of which ten incomes corresponded to one of ten income brackets within the 50th–99th percentiles, one to the income of the richest person in the *control* condition, and one to the income of the richest person in the *top 1% treatment* condition. Participants were asked to indicate their feelings (i) toward an individual with such income and (ii) when imagining an income of that amount, rating the intensity of their feelings on a sliding scale ranging from 0 (“it has little emotional effect on me”) to 100 (“I have very intense feelings”). Following our preregistration, we created an individual-level affective response score at each level of income by averaging participants’ responses to these two items. The preregistration of Study 3b is available at https://aspredicted.org/57N_RWP.

We recruited participants via Prolific. 465 respondents (50.3% female, *M*_age_ = 38.06 years) passed the attention check and completed the study. Replicating our earlier studies, we found that participants in the top 1% treatment condition underestimated the average income of the top 20% (*P* < 0.001, *Z* = −6.004, *r* = 0.404). However, we did not find evidence in support of underestimation for participants in the top 10% treatment condition (in fact, if anything, they overestimated these incomes; *P* < 0.001, *Z* = 3.661, *r* = 0.233). This pattern of results suggests that the underestimation of incomes held by the richest is unique to the top of income distribution. These findings hold subject to a number of robustness checks, detailed in the Supplementary Materials.

**Fig. 3.**
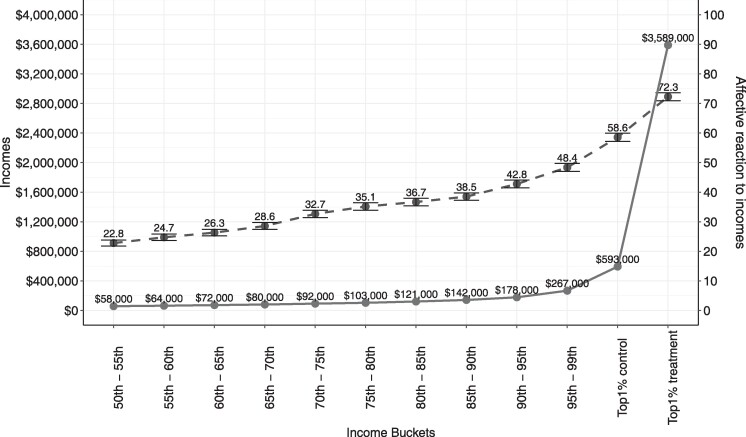
Participants’ affective response to incomes at varying levels from Study 2b. As incomes (solid line) increase, participants’ affective response (dashed line) does not increase commensurately—in line with predictions from scope insensitivity—providing suggestive evidence that this in part underlies the underestimation of the top 1% of income shares.

Next, we tested participants’ affective responses to the 12 quasi-randomly generated incomes. Following our preregistration, we aggregated all responses to each level of income and subsequently plotted affective responses against absolute incomes, displayed in Figure 3. At lower absolute income levels, we find that an increase from $58,000 to roughly twice that to $121,000 is associated with a 13.9-point increase in affective response (rated on a scale of 1–100). At the top of the income distribution, however, a nearly similar increase in affective response—13.7-point increase—occurs when moving from $593,000 to more than six times that to $3,589,000. That is, as income levels increase, participants’ affective response does not proportionally vary in response to differences in income (see also Supplementary Materials). As these findings show, participants are less sensitive to changes in incomes as absolute incomes numbers become greater, consistent with scope insensitivity.

## Discussion

The current research sought to examine whether people are uniquely insensitive to the incomes of individuals at the upper end of the income distribution. Studies 1a and 1b provided correlational support, including by incentivizing accurate responses, that respondents systematically underestimate incomes of the top 1% of the income distribution. Indeed, this insensitivity was a defining feature of the top of the income distribution but did not extend consistently and commensurably to other income percentiles. In Studies 2a and 2b, we replicated these findings in an experimental setting, finding that this selective underestimation emerges even in the absence of systemic factors, suggesting the role cognitive processes may play in contributing to this misperception. Note that while prior research finds that participants often misperceive the level of inequality in society (8–12)—sometimes finding evidence in support of underestimation (8, 14, 15) and at other times for overestimation (11, 16)—our studies provide converging evidence that participants selectively underestimate the amount of income held by the richest individuals in the population. However, our results are less clear on whether participants also misperceive incomes held by lower-income individuals.^b,c^ Taken together, our findings suggest that people systematically underestimate the extent of inequality in society.

With inequality's rise in many developed countries being largely driven by the outsized gains of the richest individuals, our results suggest that the unique underestimation of the incomes held by the upper end of the income distribution may represent a significant barrier to action (37, 38). Consider the corollary prediction that—because we find people to be insufficiently sensitive to inequality among the top 1%—their preferences for redistribution may also be less sensitive to changes among the concentration of incomes held by the top 1%. We tested this prediction in a supplementary study (SS1, see details in the Supplementary Materials) using data from the International Social Survey Program (39), which asked participants to indicate their preferences for increased taxes on high-income individuals, reflective of support for redistribution (40). We examined whether changes in the actual concentration of incomes held by the top 1% between 1999 and 2009—obtained from the World Inequality Database (41)—were related to support for redistribution, but did not find a significant effect (*b* = 0.002, SE = 0.004, 95% CI*_%_* = [-0.006, 0.010], *P* = 0.643). This result suggests that when growing inequality is driven by the income gains at the very top of the distribution, it may not necessarily be reflected in people's preferences for redistribution.

Both the upward social comparison hypothesis and concurrent overrepresentation of rich individuals in the media suggest that people may be more accurate in estimating the incomes of the richest individuals compared to those in lower-income groups. Our findings stand in contrast to these predictions and show that people tend to *under*estimate the incomes of the richest, while providing less biased estimates for those in lower income percentiles. The fact that we observed less precise estimates and underestimation for the richest does not mean that the drivers leading to more accurate estimates or overestimation are not present, but instead that they are likely less dominant than the drivers leading to underestimation.

It is likely that several factors, including both cognitive and systemic factors, jointly influence people's judgments about the upper part of the income distribution (e.g. 42, 43). For example, according to the network hypothesis, higher-income individuals have more exposure to top-income individuals and may therefore be less likely to underestimate the income of the top 1%. Indeed, in an additional analysis of data from Studies 1 and 1b, we find that participants who themselves are in the top 10% of incomes are also more accurate in their perceptions of top 1% individuals in comparison with all other participants (*P* = 0.003, *Z* = 2.973, *r* = 0.122 for Study 1a and *P* < 0.001, *Z* = 3.703, *r* = 0.139 for Study 1b). However, even in this subset of the population, there is a remaining underestimation of the top 1%, suggesting that other mechanisms likely continue to be implicated. In fact, in Studies 2a and 2b, we were able to remove the potential influence of systemic barriers and the potential for network effects through our experimental designs and continued to find a unique underestimation of the top 1%, suggesting that cognitive biases also contribute to this effect.

Our pattern of results—the increasing underestimation at the upper echelons as we ascend in the income distribution—is consistent with selective scope insensitivity at the very top of the income distribution. The mechanisms underlying scope insensitivity suggest that instead of the quantity of a target object (e.g. the exact amount of dollars owned by a rich person), the affect triggered by the prototypical image of the object (e.g. a rich person) conveys meaning to individuals (27, 31, 32). As a result, an additional amount of income in upper echelons of the income distribution may trigger a weaker affective response than the same amount of additional income in lower parts of the income distribution. That is, from the perspective of an observer, a person earning $1.3 million—the average income of the richest 1% of the US population (44)—is seen as less different from a person earning $1.4 million than the same absolute difference at lower income levels, for instance, between $50,000 and $150,000$. This is exactly what we observed in Study 2b, where participants’ affective response to different income levels did not proportionally vary in response to the differences in income.

However, we make clear that this evidence is not definitive evidence in favor of scope insensitivity. One potential constraint on our interpretation of these findings is that incomes are theoretically unconstrained (i.e. incomes can go higher and higher), whereas we bounded the scale for participants’ emotional response between 0 and 100. That is, it is possible that study participants opted to wait for a potentially higher income that never came, and which therefore may in part contribute to our observed pattern of results. While our evidence suggests a plausible cognitive mechanism underlying the observed pattern of results, future research could investigate whether other cognitive processes, including the underweighting of rare events in the averaging process (e.g. 35) or magnitude neglect (e.g. 26) may also contribute to the selective underestimation we observe here.

Our findings also suggest that it is unclear whether correcting perceptions about the income of the top 1% may necessarily lead to a significant change in redistributive preferences. Consider that prior intervention studies which seek to provide people with accurate information about incomes along the income distribution either do not significantly affect support for redistribution (6) or only weakly do so (40). It is possible that the presence of cognitive biases—in line with the current research—is particularly pernicious and difficult-to-intervene on, complicating progress toward increased support for redistribution (25).^d^

A limitation of our findings is that all reported studies collected data from US participants, raising the question whether our findings are generalizable to other populations. We provide preliminary evidence that our main findings may also replicate around the world in the same supplementary study referred to above (SS1, see detailed methods reported in the Supplementary Materials; *N*_1999_ = 23,288 and *N*_2009_ = 51,970), where we used participants’ income estimates for different occupations to approximate levels of perceived inequality in 19 and 40 countries for 1999 and 2009 which we compared to objective income data from the World Inequality Database. While the results of this supplementary study are in line with our main findings, we urge caution in viewing these as conclusive data given rough occupational estimates and encourage future research to further investigate the robustness of these results.

Another limitation of our study is that we are unable to pinpoint the extent to which the geographical scale used during the elicitation in Studies 1a and 1b (i.e. the county level) impacted our results. It is possible that at different geographical scales (e.g. the state level), people may rely on different cues, such as on information from the media, when making their estimations, which could potentially lead to different results—a possibility we encourage future research to examine. Finally, we acknowledge that asking people to make numeric estimates may not necessarily map on to how they experience and respond to inequality in everyday life (see e.g. 10, 36). As a result, we encourage future research to adopt a broader variety of methods, including nonnumerical and more experiential measures, to examine whether the selective underestimation of the incomes of the richest which we suggest contributes to the underestimation of inequality more broadly also manifests in the way people commonly think about and respond to inequality.

## Conclusion

In sum, our theory and findings suggest that people are uniquely insensitive to the incomes held by individuals at the top of the income distribution. This takes on particular importance given that increases in inequality in many developed countries are often located in the disproportionate growth of incomes among the top 1% (1–3). While the recognition of inequality is commonly viewed as a critical factor in increasing support for redistributive policies (e.g. 46–49), the current research highlights how cognitive biases may complicate people's recognition of inequality particularly among the richest—and thereby impede progress for more redistributive policies (32). Beyond summary measures of inequality, research and policy may also need to shift toward being more sensitive to where inequality is concentrated (50).

## Materials and methods

### Study 1a

Only current US residents and those who passed the attention check were able to participate and complete the survey. Respondents were paid US$1.30 for their participation. To increase data quality, we excluded inattentive participants, defined as those who answered 0 or 1 for every estimate, who did not write monotonically increasing income distributions, and who indicated distributions that were not realistically possible. That is, we excluded distributions that only had one increase throughout the distribution (e.g. 10, 10, 10, 10, 10, 20, 20, 20, 20, 20, 20), had no increase in the distributions (e.g. 10, 10, 10, 10, 10, 10, 10, 10, 10, 10, 10), had “0” for at least four percentiles (e.g. 0, 0, 0, 0, 10, 20, 30, 40, 50, 60), had a one-point increase for all percentiles (e.g. 1, 2, 3, 4, 5, 6, 7, 8, 9, 10, 11), or reflected the percentiles themselves (e.g. 10, 20, 30, 40, 50, 60, 70, 80, 90, 95, 99). See Supplementary Materials for the number of distributions that we excluded for each category. The final sample size after these exclusions consisted of 593 participants (54.8% female, *M*_age_ = 46.59). Note that the analysis without any exclusions yielded qualitatively congruent results. The full survey, the calculation of the objective thresholds for each county, the detailed results for the clustered Wilcoxon tests in each percentile, and the robustness tests are available in the Supplementary Materials. Study 1a was approved by the Institutional Review Board of Harvard Business School (IRB20-1176). We confirm that informed consent was obtained from all participants.

### Study 1b

Respondents were paid a base reimbursement of US$1.30 for their participation. We applied the same exclusion criteria as in Study 1a. The sample size after exclusions consisted of 711 participants (47.12% female, *M*_age_ = 36.18). The income shares were calculated identically to Study 1a. The full survey, calculation of the objective thresholds for each county, detailed results for the clustered Wilcoxon tests in each percentile, and robustness tests are available in the Supplementary Information.

The analyses testing the effect of wagering on the accuracy of the estimations were not part of the preregistration. In the preregistration, we indicated that we aimed to recruit 1,000 participants but we decided to stop data collection by 934 as the rate of new individuals willing to participate in the study became so low that it was unrealistic to reach our target number within a sensible timeframe. We did not analyze the data before making the decision to stop data collection. To make more conservative estimates, we used two-sided tests for all of the analyses. Study 1b was approved by the Institutional Review Board of Harvard Business School (IRB21-0364). We confirm that informed consent was obtained from all participants.

### Studies 2a and 2b

The presentation order of the control and treatment condition was randomly assigned and counterbalanced across participants. The distribution of presented incomes was matched with data provided by the US Census Bureau with respect to the mean and the percentage of people belonging to different income brackets. To do so, we pooled some of the income brackets while we calculated the weighted mean and the total proportion of people in the new income brackets. Afterward, we randomly generated two sets of numbers with the mean of each income bracket. The number of generated incomes was proportional to the percentage of people in the given income bracket. The presentation of the two societies with different income values was counterbalanced between the two conditions, and their order was randomized. The exact income distributions are given in the Supplementary Materials. The position of the richest individual in the top 1% treatment condition varied and was counterbalanced between the 5th, 25th, 50th, 75th, and 95th positions, while the position of the 2–10% of individuals in the top 10% was randomly assigned during the presentation.

Measuring perceived inequality can be sensitive to the units of measurements (e.g. 33, 45). Eriksson and Simpson (33) provided evidence that asking participants to express their estimates as averages instead of proportions leads to more accurate responses. Accordingly, after the presentation of the income distributions, we asked participants the following questions: *What do you think is the average income of the richest/second/middle/fourth*/*poorest 20% of the individuals in the presented society?* The objective income average for each quintile was calculated from the true values of the distributions.

In both Studies 2a and 2b, respondents were paid US$2.40 base reimbursement for their participation. The full surveys, the incomes presented in Studies 2a and 2b, the detailed results for the Wilcoxon tests in each quintile, and the robustness tests are all available in the Supplementary Materials. As opposed to the preregistration, we used two-sided tests for all the analyses to make more conservative conclusions. Studies 2a and 2b were approved by the Institutional Review Board of Harvard Business School (IRB21-1123). We confirm that informed consent was obtained from all participants.

## Supplementary Material

pgae333_Supplementary_Data

## Data Availability

The HTML code of the experiments, complete surveys and materials, the anonymized data, the preprocessing, and the analysis codes for each study are publicly available on the Open Science Framework (https://osf.io/hszyp/). Data for Study 2 are available for download at https://issp.org/data-download/by-year/.

## Notes

a For participants who did not know their county, we provided an easy lookup tool that cross-referenced their county matched by their zip code.

b Note that in contrast to our findings, Chambers et al (16). found in their Study 3 that participants overestimated the cutoff for the top 1% of earners. It would be interesting to investigate the potential reasons behind these disparities (e.g. Chambers et al. studied the reported cutoff points in a two-choice paradigm while we asked participants to freely indicate any number indicating the average incomes; for a more detailed discussion, see also Jachimowicz et al. (12)).

c While this was not a focus of our research, we also recommend future research to further explore why people sometimes overestimate the incomes held by lower-income percentiles. For instance, it is possible that these reflect system justifying beliefs, i.e. that people hope that lower-income individuals are richer than they really are (e.g. in line with Kai and Jost (35) and Ward and King (36)).

d A related mechanism may be whether participants think about specific individuals who may be part of the top 1% income earners, or more abstractly think about top 1% income groups. Consider that in one of their studies, Chambers et al. (16) asked participants to estimate the number of people in very-high income buckets (i.e. $1m to $4.9m, $5m and above), and find that participants overestimated how many people in the United States are within each bucket. In contrast, Walker et al. (45) show that participants are more tolerant of inequality when focusing on specific individuals who are at the top of the income distribution, in contrast to the top 1% as a group. Given these mixed findings, we encourage future research to further disentangle how the perception of inequality may differ when focusing on specific individuals versus the top 1% in society more broadly.

## Deviations from the Preregistration

Our preregistration specified that we would perform all analyses based on income shares. Following the suggestion of an anonymous reviewer, we opted to conduct analyses for all of our studies directly on the nontransformed data, i.e. not converting estimated thresholds into income shares. We made this change for several reasons: (i) converting to income shares could potentially yield different results depending on the measure used, (ii) it might create an artificial underestimation in the lower percentiles, and (iii) it could bias the results by extrapolating based on potentially questionable assumptions. Importantly, both the originally planned and preregistered analyses, and this different way of analyzing the data lead to qualitatively equivalent conclusions.

## References

[1] Saez E, Zucman G. 2016. Wealth inequality in the United States since 1913: evidence from capitalized income tax data. Q J Econ. 131(2):519–578.

[2] Smith M, Zidar O, Zwick E. 2022. Top wealth in America new estimates under heterogeneous returns. Q J Econ. 138(1):515–573.

[3] Saez E, Zucman G. 2020. The rise of income and wealth inequality in America: evidence from distributional macroeconomic accounts. J Econ Perspect. 34(4):3–26.

[4] Peyton K . 2020. Does trust in government increase support for redistribution? Evidence from randomized survey experiments. Am Polit Sci Rev. 114(2):596–602.

[5] Bechtel MM, Liesch R, Scheve KF. 2018. Inequality and redistribution behavior in a give-or-take game. Proc Natl Acad Sci U S A. 115(14):3611–3616. 29555734 10.1073/pnas.1720457115 PMC5889654

[6] Ciani E, Fréget L, Manfredi T. 2021. Learning about inequality and demand for redistribution: a meta-analysis of in-survey informational experiments. 10.1787/8876ec48-en

[7] OECD . 2021. Does inequality matter? How people perceive economic disparities and social mobility.

[8] Norton MI, Ariely D. 2011. Building a better America—one wealth quintile at a time. Perspect Psychol Sci. 6(1):9–12. 26162108 10.1177/1745691610393524

[9] Hauser OP, Norton MI. 2017. (Mis)perceptions of inequality. Curr Opin Psychol. 18:21–25. 29221507 10.1016/j.copsyc.2017.07.024

[10] Phillips TL, et al 2022. Inequality in People's Minds, Working paper.

[11] Gimpelson V, Treisman D. 2018. Misperceiving inequality. Econ Polit. 30(1):27–54.

[12] Jachimowicz JM, et al 2022. Inequality in researchers’ minds: four guiding questions for studying subjective perceptions of economic inequality. J Econ Surv. 37(5):1534–1561.

[13] Hauser OP, Kraft-Todd GT, Rand DG, Nowak MA, Norton MI. 2021. Invisible inequality leads to punishing the poor and rewarding the rich. Behav Public Policy. 5(3):333–353.

[14] Kiatpongsan S, Norton MI. 2014. How much (more) should CEOs make? A universal desire for more equal pay. Perspect Psychol Sci. 9(6):587–593. 26186109 10.1177/1745691614549773

[15] Kraus MW, Onyeador IN, Daumeyer NM, Rucker JM, Richeson JA. 2019. The misperception of racial economic inequality. Perspect Psychol Sci. 14(6):899–921. 31505132 10.1177/1745691619863049

[16] Chambers JR, Swan LK, Heesacker M. 2014. Better off than we know: distorted perceptions of incomes and income inequality in America. Psychol Sci. 25(2):613–618. 24317422 10.1177/0956797613504965

[17] Payne BK, Brown-Iannuzzi JL, Hannay JW. 2017. Economic inequality increases risk taking. Proc Natl Acad Sci U S A. 114(18):4643–4648. 28416655 10.1073/pnas.1616453114 PMC5422783

[18] Kim E . 2019. Entertaining beliefs in economic mobility. Am J Polit Sci. 67(1):39–54.

[19] Dawtry RJ, Sutton RM, Sibley CG. 2015. Why wealthier people think people are wealthier, and why it matters: from social sampling to attitudes to redistribution. Psychol Sci. 26(9):1389–1400. 26187249 10.1177/0956797615586560

[20] Feiler DC, Kleinbaum AM. 2015. Popularity, similarity, and the network extraversion bias. Psychol Sci. 26(5):593–603. 25838113 10.1177/0956797615569580

[21] Dawtry RJ, Sutton RM, Sibley CG. 2019. Social sampling, perceptions of wealth distribution, and support for redistribution. In: Jetten J, Peters K, editors. The social psychology of inequality. Cham: Springer. p. 381–396.

[22] Sands ML, de Kadt D. 2020. Local exposure to inequality raises support of people of low wealth for taxing the wealthy. Nature. 586(7828):257–261. 32968274 10.1038/s41586-020-2763-1

[23] Waldfogel HB, Sheehy-Skeffington J, Hauser OP, Ho AK, Kteily NS. 2021. Ideology selectively shapes attention to inequality. Proc Natl Acad Sci U S A. 118(14):e2023985118. 33795517 10.1073/pnas.2023985118 PMC8040796

[24] Bogard EJ, West C, Fox CR. 2022. Heuristics and biases in evaluations of economic inequality. Working Paper. 10.21203/rs.3.rs-1278751/v1

[25] Jackson JC, Payne K. 2021. Cognitive barriers to reducing income inequality. Soc Psychol Personal Sci. 12(5):687–696.

[26] Boyce-Jacino C, Peters E, Galvani AP, Chapman GB. 2022. Large numbers cause magnitude neglect: the case of government expenditures. Proc Natl Acad Sci U S A. 119(28):e2203037119. 35867746 10.1073/pnas.2203037119 PMC9282355

[27] Fetherstonhaugh D, Slovic P, Johnson S, Friedrich J. 1997. Insensitivity to the value of human life: a study of psychophysical numbing. J Risk Uncertain. 14(3):283–300.

[28] Dickert S, Västfjäll D, Kleber J, Slovic P. 2015. Scope insensitivity: the limits of intuitive valuation of human lives in public policy. J Appl Res Mem Cogn. 4(3):248–255.

[29] Von Neumann J, Morgenstern O. 2007. Theory of games and economic behavior. Princeton, New Jersey: Princeton University Press.

[30] Fechner GT . 1966. Elements of psychophysics. New York: Holt, Rinehart & Winston. (Original work published 1860).

[31] Sommeiller E, Price M, Wazeter E. 2016. Income inequality in the U.S. by state, metropolitan area, and county. Washington: Econ Policy Inst.

[32] Blesch K, Hauser OP, Jachimowicz JM. 2022. Measuring inequality beyond the Gini coefficient may clarify conflicting findings. Nat Hum Behav. 6(11):1525–1536. 36038775 10.1038/s41562-022-01430-7 PMC7614289

[33] Eriksson K, Simpson B. 2012. What do Americans know about inequality? It depends on how you ask them. Judgm Decis Mak. 7(6):741–745.

[34] Mummolo J, Peterson E. 2019. Demand effects in survey experiments: an empirical assessment. Am Polit Sci Rev. 113(2):517–529.

[35] Hertwig R, Barron G, Weber EU, Erev I. 2004. Decisions from experience and the effect of rare events in risky choice. Psychol Sci. 15(8):534–539. 15270998 10.1111/j.0956-7976.2004.00715.x

[36] Willis GB, García-Sánchez E, Sánchez-Rodríguez Á, García-Castro JD, Rodríguez-Bailón R. 2022. The psychosocial effects of economic inequality depend on its perception. Nat Rev Psychol. 1(5):301–309.

[37] Trump K-S, White A. 2018. Does inequality beget inequality? Experimental tests of the prediction that inequality increases system justification motivation. J Exp Polit Sci. 5(3):206–216.

[38] Trump K-S . 2018. Income inequality influences perceptions of legitimate income differences. Br J Polit Sci. 48(4):929–952.

[39] ISSP Research Group . 2022. International Social Survey Programme: Social Inequality V—ISSP 2019. GESIS, Cologne. ZA7600 Data file Version 3.0.0.

[40] Kuziemko I, Norton MI, Saez E, Stantcheva S. 2015. How elastic are preferences for redistribution? Evidence from randomized survey experiments. Am Econ Rev. 105(4):1478–1508.

[41] Chancel L, et al World Inequality Report 2022. World Inequality Lab.

[42] Chetty R, Hendren N. 2018. The impacts of neighborhoods on intergenerational mobility II: county-level estimates. Q J Econ. 133(3):1163–1228.

[43] Chetty R, et al 2022. Social capital I: measurement and associations with economic mobility. Nature. 608(7921):108–121. 35915342 10.1038/s41586-022-04996-4 PMC9352590

[44] Piketty T, Saez E, Zucman G. 2018. Distributional national accounts: methods and estimates for the United States. Q J Econ. 133(2):553–609.

[45] Heiserman N, Simpson B. 2021. Measuring perceptions of economic inequality and justice: an empirical assessment. Soc Justice Res. 34(2):119–145.

[46] Engelhardt C, Wagener A. 2014. Bi ased perceptions of income inequality and redistribution. Working paper.

[47] Koster R, et al 2022. Human-centred mechanism design with democratic AI. Nat Hum Behav. 6(10):1398–1407. 35789321 10.1038/s41562-022-01383-x PMC9584820

[48] Hauser OP, Hilbe C, Chatterjee K, Nowak MA. 2019. Social dilemmas among unequals. Nature. 572(7770):524–527. 31413366 10.1038/s41586-019-1488-5

[49] Meltzer AH, Richard SF. 1983. Tests of a rational theory of the size of government. Public Choice. 41(3):403–418.

[50] Tan J, Jachimowicz J, Smerdon D, Hauser O. 2020. Opposing effects of economic inequality concentrated at the top or bottom of the income distribution on subjective well-being. Working paper.

