# Supplementary material for: Selective insensitivity to income held by the richest
Source: PNAS Nexus. 2024 Sep 17;3(9):pgae333. doi: 10.1093/pnasnexus/pgae333 (PMC11407284; doi:10.1093/pnasnexus/pgae333)
Supplement: pgae333_Supplementary_Data [file pgae333_supplementary_data.docx]

**Supplementary Materials for**

**Selective insensitivity to income held by the richest**

The implementation code of the experiments, the complete surveys, the data, the pre-processing, and the analysis codes for each study are available at the project’s OSF page: <https://osf.io/hszyp/>**.**

**Study 1a and Study 1b**

**Table S1.** Under- and Overestimation of income thresholds of different percentiles (Study 1a)

| Percentile | Median Underestimation | SD Underestimation | *P* | *Z* | Effect Size (r) |
| --- | --- | --- | --- | --- | --- |
| 10^th^ | -1465.892 | 14543.869 | < .001 | -5.359 | .220 |
| 20^th^ | 985.860 | 18611.127 | .003 | 2.933 | .120 |
| 30^th^ | 3158.069 | 24206.957 | < .001 | 5.688 | .234 |
| 40^th^ | 5939.981 | 31640.099 | < .001 | 6.872 | .282 |
| 50^th^ | 6858.308 | 41988.005 | < .001 | 7.490 | .308 |
| 60^th^ | 8178.731 | 60710.240 | < .001 | 7.495 | .308 |
| 70^th^ | 13358.516 | 90116.756 | < .001 | 8.277 | .340 |
| 80^th^ | 22654.150 | 424605.116 | < .001 | 9.717 | .399 |
| 90^th^ | 49447.327 | 4110765.769 | < .001 | 11.976 | .492 |
| 95^th^ | 81406.991 | 41065679.232 | < .001 | 12.358 | .507 |
| 99^th^ | 207575.000 | 582973743.371 | < .001 | 12.490 | .513 |

**Table S2.** Under- and Overestimations of income thresholds of different percentiles (Study 1a without exclusions)

| Percentile | Median Underestimation | SD Underestimation | *P* | *Z* | Effect Size (r) |
| --- | --- | --- | --- | --- | --- |
| 10^th^ | -330.405 | 4546942.907 | < .001 | -4.515 | .143 |
| 20^th^ | 4094.773 | 485225.980 | < .001 | 3.717 | .118 |
| 30^th^ | 6896.192 | 694633.423 | < .001 | 5.862 | .186 |
| 40^th^ | 10063.282 | 451466.750 | < .001 | 8.491 | .270 |
| 50^th^ | 12752.058 | 601834.837 | < .001 | 11.160 | .355 |
| 60^th^ | 19411.067 | 2701528572.594 | < .001 | 13.714 | .436 |
| 70^th^ | 30388.760 | 2860504033.248 | < .001 | 15.607 | .496 |
| 80^th^ | 45408.089 | 167461011471.416 | < .001 | 17.342 | .551 |
| 90^th^ | 83585.097 | 37675729569.459 | < .001 | 19.409 | .617 |
| 95^th^ | 125953.623 | 12635465773.856 | < .001 | 19.299 | .613 |
| 99^th^ | 264413.000 | 1059759566445700.000 | < .001 | 19.602 | .623 |

**Figure S1.** Bourdon test (Study 1b)
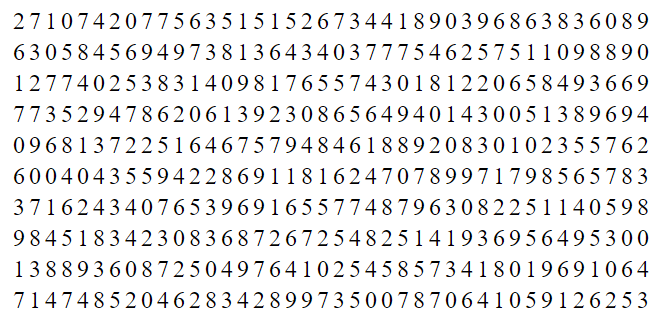


The numbers presented in Bourdon test in Study 1b. Participants were asked to count the amount of “2”s present in the figure.

**Table S3.** Under- and Overestimation of income thresholds of different percentiles (Study 1b)

| Percentile | Median Underestimation | SD Underestimation | *P* | *Z* | Effect Size (r) |
| --- | --- | --- | --- | --- | --- |
| 10^th^ | -4471.481 | 13492.103 | < .001 | -12.579 | .472 |
| 20^th^ | -2489.944 | 15177.161 | < .001 | -5.473 | .205 |
| 30^th^ | -1799.012 | 19034.913 | .009 | -2.629 | .099 |
| 40^th^ | -1921.157 | 24340.042 | .118 | -1.562 | .059 |
| 50^th^ | -2254.291 | 33332.304 | .134 | -1.499 | .056 |
| 60^th^ | -2052.845 | 49602.462 | .248 | -1.156 | .043 |
| 70^th^ | -883.758 | 70073.189 | .541 | -0.612 | .023 |
| 80^th^ | 2293.734 | 105256.702 | .514 | 0.653 | .024 |
| 90^th^ | 20062.399 | 213219.987 | < .001 | 4.755 | .178 |
| 95^th^ | 40874.955 | 1055742.321 | < .001 | 6.813 | .256 |
| 99^th^ | 129483.000 | 37519565.108 | < .001 | 7.520 | .282 |

**Table S4.** Under- and Overestimation of income thresholds of different percentiles (Study 1b without exclusions)

| Percentile | Median Underestimation | SD Underestimation | *P* | *Z* | Effect Size (r) |
| --- | --- | --- | --- | --- | --- |
| 10^th^ | -5050.133 | 316448.993 | < .001 | -14.968 | .521 |
| 20^th^ | -2924.953 | 98288.842 | < .001 | -7.899 | .275 |
| 30^th^ | -2633.358 | 48603.715 | < .001 | -5.173 | .180 |
| 40^th^ | -2769.227 | 102425.678 | < .001 | -3.598 | .125 |
| 50^th^ | -2396.830 | 39665.900 | .032 | -2.147 | .075 |
| 60^th^ | -933.305 | 64624.215 | .736 | -0.337 | .012 |
| 70^th^ | 962.631 | 76791.173 | .228 | 1.206 | .042 |
| 80^th^ | 5480.956 | 201187.525 | .004 | 2.895 | .101 |
| 90^th^ | 26270.027 | 219009.611 | < .001 | 7.042 | .245 |
| 95^th^ | 51730.936 | 1002118.961 | < .001 | 8.858 | .309 |
| 99^th^ | 155526.000 | 34855854.377 | < .001 | 9.627 | .335 |

*Analysis of betting behavior and prediction accuracy (Study 1b)*

In total, 529 of the 711 participants (74%) wagered at least some money, suggesting that most participants were at least somehwat confident about their estimates regarding the income thresholds in their county (Figure S2, *Mdn* = $0.25, *Mean* = $0.23).

**Figure S2.** Histogram of bets wagered (Study 1b)


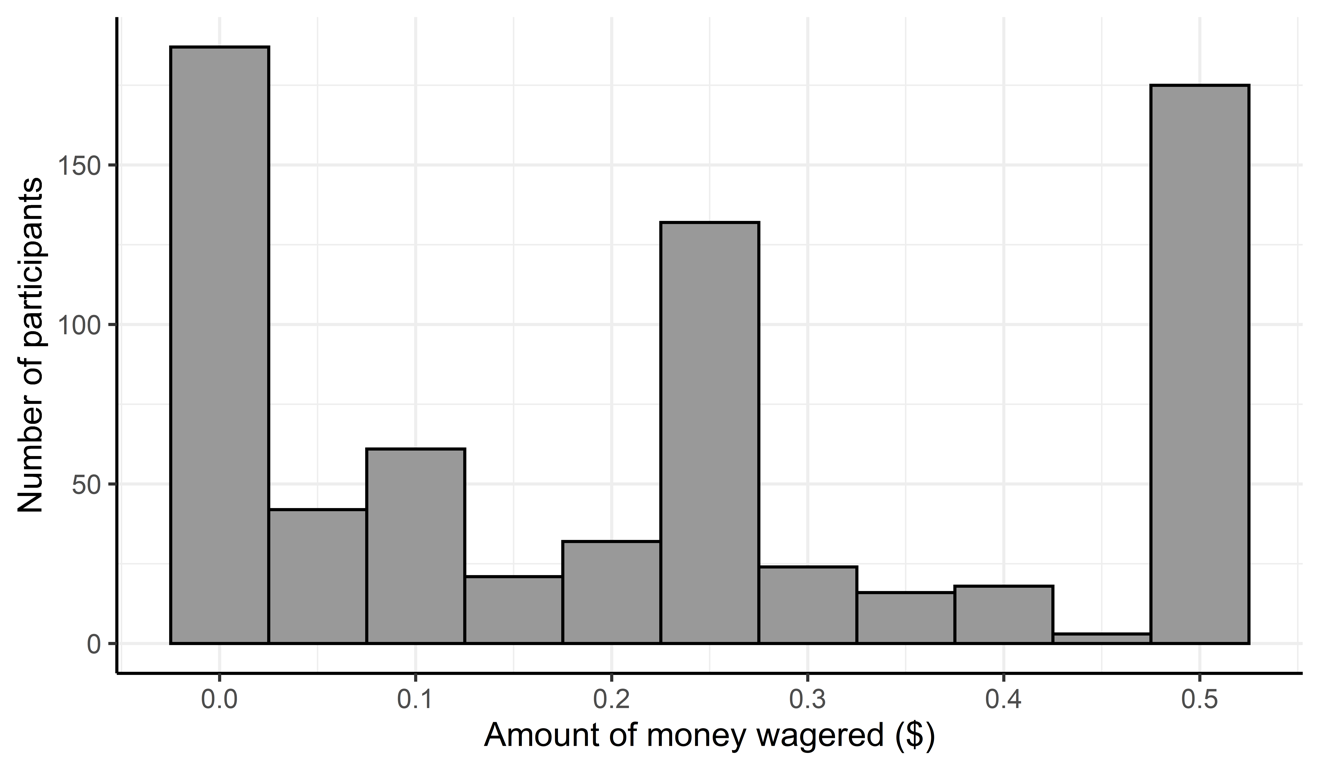


We compared the accuracy of estimates between participants who wagered all of their additional earnings ($0.50) and participants who did not wager any of their earnings ($0). As seen in Figure S3, participants who wagered all $0.50 of their earnings were no more accurate than those who wagered none of their earnings (Tables S5 and S6). These results suggest that even those who were highly confident of their estimates (and presumably even more motivated to get their estimates right) also underestimated the top percentiles to a similar extent as those who were less confident.

**Figure S3.** Estimates of those who wagered all of their bonus (top) and of those who wagered none of their bonus (bottom) show a similar pattern (Study 1b)


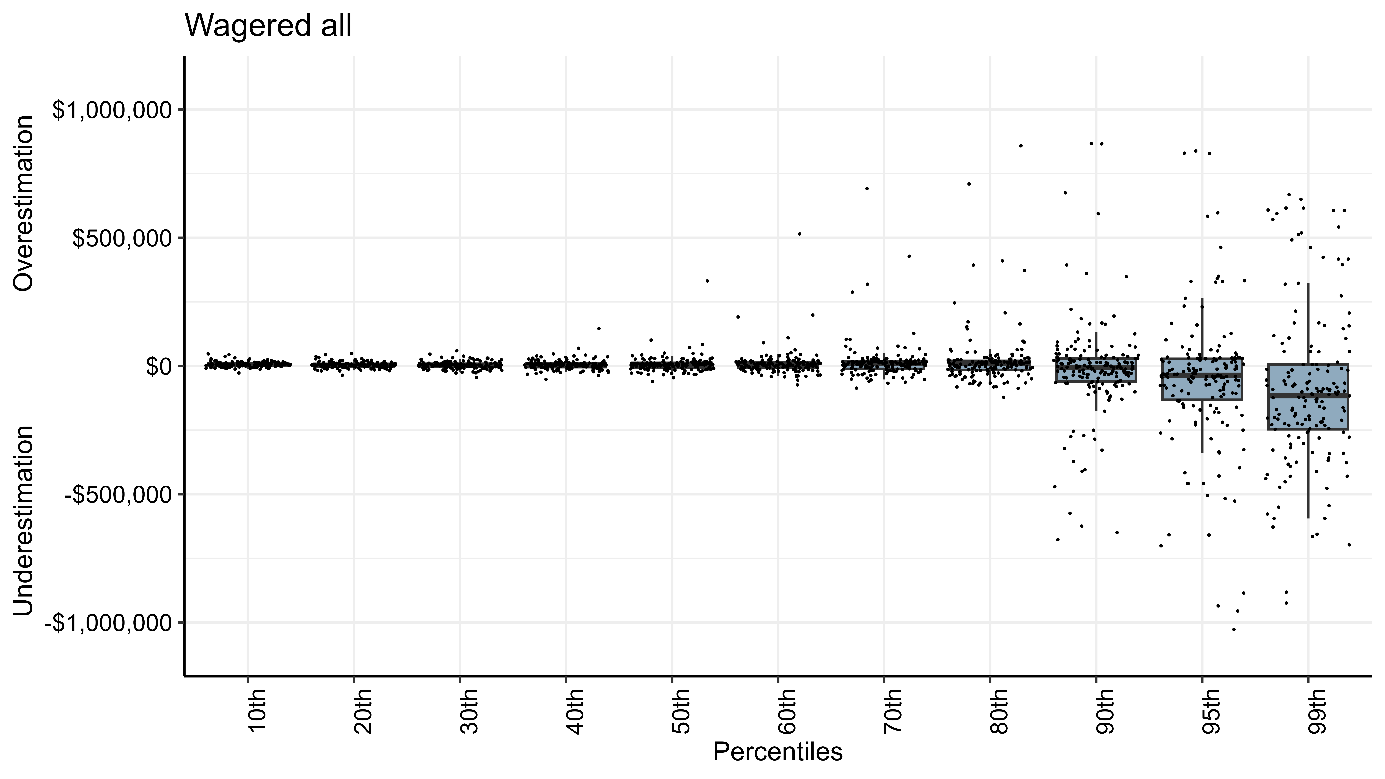

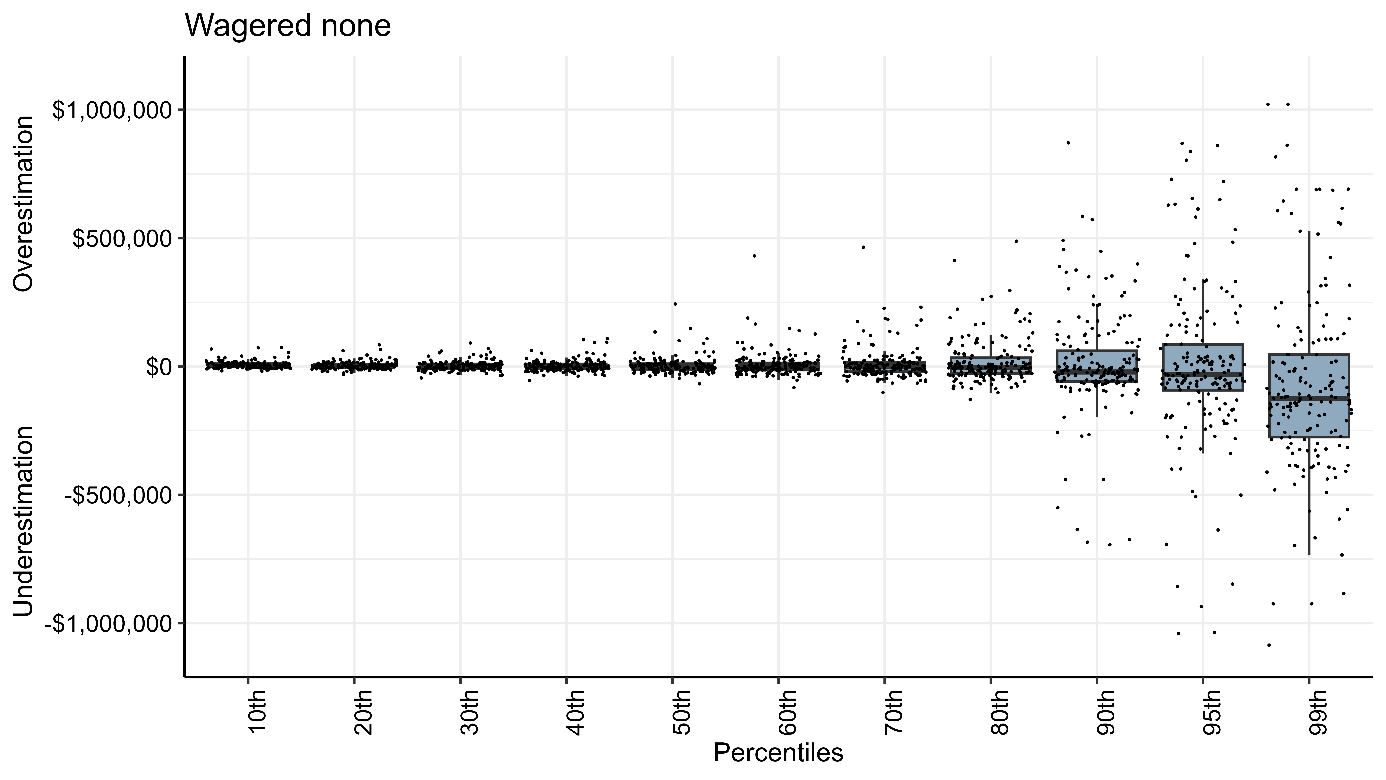


**Table S5.** Under- and Overestimation of income thresholds of different percentiles for participants who wagered all of their earnings (Study 1b)

| Percentile | Median Underestimation | SD Underestimation | *P* | *Z* | Effect Size (r) |
| --- | --- | --- | --- | --- | --- |
| 10^th^ | -4900.686 | 9822.064 | < .001 | -7.524 | .572 |
| 20^th^ | -3276.465 | 11366.939 | < .001 | -4.666 | .355 |
| 30^th^ | -3334.221 | 13639.216 | < .001 | -3.395 | .258 |
| 40^th^ | -3681.021 | 19086.744 | < .001 | -3.367 | .256 |
| 50^th^ | -4636.830 | 32123.948 | < .001 | -3.343 | .254 |
| 60^th^ | -7795.086 | 49153.191 | < .001 | -3.331 | .253 |
| 70^th^ | -8674.106 | 74931.641 | .003 | -2.946 | .224 |
| 80^th^ | -8754.682 | 108686.454 | .044 | -2.012 | .153 |
| 90^th^ | 4922.370 | 233573.908 | .136 | 1.489 | .113 |
| 95^th^ | 32887.240 | 776215.510 | < .001 | 3.754 | .285 |
| 99^th^ | 109882.000 | 1989108.250 | < .001 | 3.513 | .267 |

**Table S6.** Under- and Overestimation of income thresholds of different percentiles for participants who wagered none of their earnings (Study 1b)

| Percentile | Median Underestimation | SD Underestimation | *P* | *Z* | Effect Size (r) |
| --- | --- | --- | --- | --- | --- |
| 10^th^ | -4592.421 | 13634.944 | < .001 | -6.491 | .481 |
| 20^th^ | -1314.624 | 14678.117 | .010 | -2.576 | .191 |
| 30^th^ | -176.484 | 17936.238 | .248 | -1.154 | .086 |
| 40^th^ | 34.214 | 22758.743 | .750 | -0.318 | .024 |
| 50^th^ | 99.007 | 32679.230 | .871 | -0.162 | .012 |
| 60^th^ | 440.490 | 48016.821 | .910 | -0.113 | .008 |
| 70^th^ | 1221.141 | 61735.319 | .761 | 0.304 | .023 |
| 80^th^ | 4022.159 | 85772.112 | .812 | -0.238 | .018 |
| 90^th^ | 21391.204 | 199661.369 | .313 | 1.008 | .075 |
| 95^th^ | 31203.881 | 1868833.847 | .254 | 1.142 | .085 |
| 99^th^ | 116503.000 | 74111938.847 | .019 | 2.337 | .173 |

**Table S7.** Comparing underestimation between 95^th^  and 99^th^ percentiles (Study 1a and 1b).

| Percentile | Mean | Median | SD | *Z* | *p* | *r* |
| --- | --- | --- | --- | --- | --- | --- |
| Study 1a |  |  |  |  |  |  |
| 95^th^ | 1633125.44 | -81406.99 | 41065679.23 | 24.623 | < .001 | .553 |
| 99^th^ | 37805651.33 | -207575.00 | 582973743.37 |  |  |  |
| Study 1b |  |  |  |  |  |  |
| 95^th^ | 25689.36 | -40874.96 | 1055742.32 | 8.439 | < .001 | .316 |
| 99^th^ | 1531015.76 | -129483.00 | 37519565.11 |  |  |  |

**Table S8.** Type of estimations excluded from the analysis (Study 1a)

| Description | Example | Number of exclusions* |
| --- | --- | --- |
| Previous percentile is higher than the next percentile | 100, 90, 80, 70, 60, 50, 40, 30, 20, 10 | 346 (0) |
| No increase in the distribution | 10, 10, 10, 10, 10, 10, 10, 10, 10, 10 | 22 (0) |
| Only one increase in the distribution | 10, 10, 10, 10, 10, 20, 20, 20, 20, 20, 20 | 7 (0) |
| Indicated “0” for at least 4 percentiles | 0, 0, 0, 0, 10, 20, 30, 40, 50, 60 | 6 (5) |
| Indicated only one point increase for all percentiles | 1, 2, 3, 4, 5, 6, 7, 8, 9, 10, 11 | 8 (0) |
| Reflected the percentiles themselves | 10, 20, 30, 40, 50, 60, 70, 80, 90, 95, 99 | 14 (1) |
|  | Total number of excluded cases | 397 (403) |

*Note.** The numbers in the brackets represent the number of cases that belong to the given type of estimations but are also counted in other categories.

**Table S9.** Type of estimations excluded from the analysis (Study 1b)

| Description | Example | Number of exclusions* |
| --- | --- | --- |
| Previous percentile is higher than the next percentile | 100, 90, 80, 70, 60, 50, 40, 30, 20, 10 | 112 (0) |
| No increase in the distribution | 10, 10, 10, 10, 10, 10, 10, 10, 10, 10 | 1 (0) |
| Only one increase in the distribution | 10, 10, 10, 10, 10, 20, 20, 20, 20, 20, 20 | 0 (0) |
| Indicated “0” for at least 4 percentiles | 0, 0, 0, 0, 10, 20, 30, 40, 50, 60 | 0 (0) |
| Indicated only one point increase for all percentiles | 1, 2, 3, 4, 5, 6, 7, 8, 9, 10, 11 | 0 (0) |
| Reflected the percentiles themselves | 10, 20, 30, 40, 50, 60, 70, 80, 90, 95, 99 | 0 (0) |
|  | Total number of excluded cases | 113 (113) |

*Note.* * The numbers in the brackets represent the number of cases that belong to the given type of estimations but are also counted in other categories.

**Testing the network hypothesis (Study 1a and 1b)**

In Study 1a and 1b, we also aimed to explore the predictions of the network hypothesis. According to this hypothesis, the less frequently individuals meet with people from other social classes, the less accurately they can predict the income of people in this social class. To test the predictions regarding the top of the income distribution, we first calculated the average income in each of the investigated income brackets in each county based on data from the Economic Policy Institute, and used this information and participants’ reported household income to pair participants to their positions in the income distribution. We used this information to calculate their objective distance from the top 1%. We assumed that the relationship between one’s objective distance from another class and the number of interactions with individuals in this social class is not necessarily linear. Consequently, we first plotted the data to explore the shape of the relationship. The results showed similar patterns for Study 1a and Study 1b (Figure S4). Poorer individuals more strongly underestimated the income threshold of the top 1% than richer individuals. In line with predictions of the network hypothesis, statistical tests comparing the predictions of individuals belonging to the 90^th^ -100^th^ percentiles vs poorer individuals supported evidence for this both in Study 1a (*p* = .003, *Z* = 2.993, *r* = .122) and Study 1b (*p* < .001, *Z* = 3.704, *r* = .139).

**Figure S4***.* Misperception of top 1%’s income threshold by participants’ own income (Study 1a and 1b)


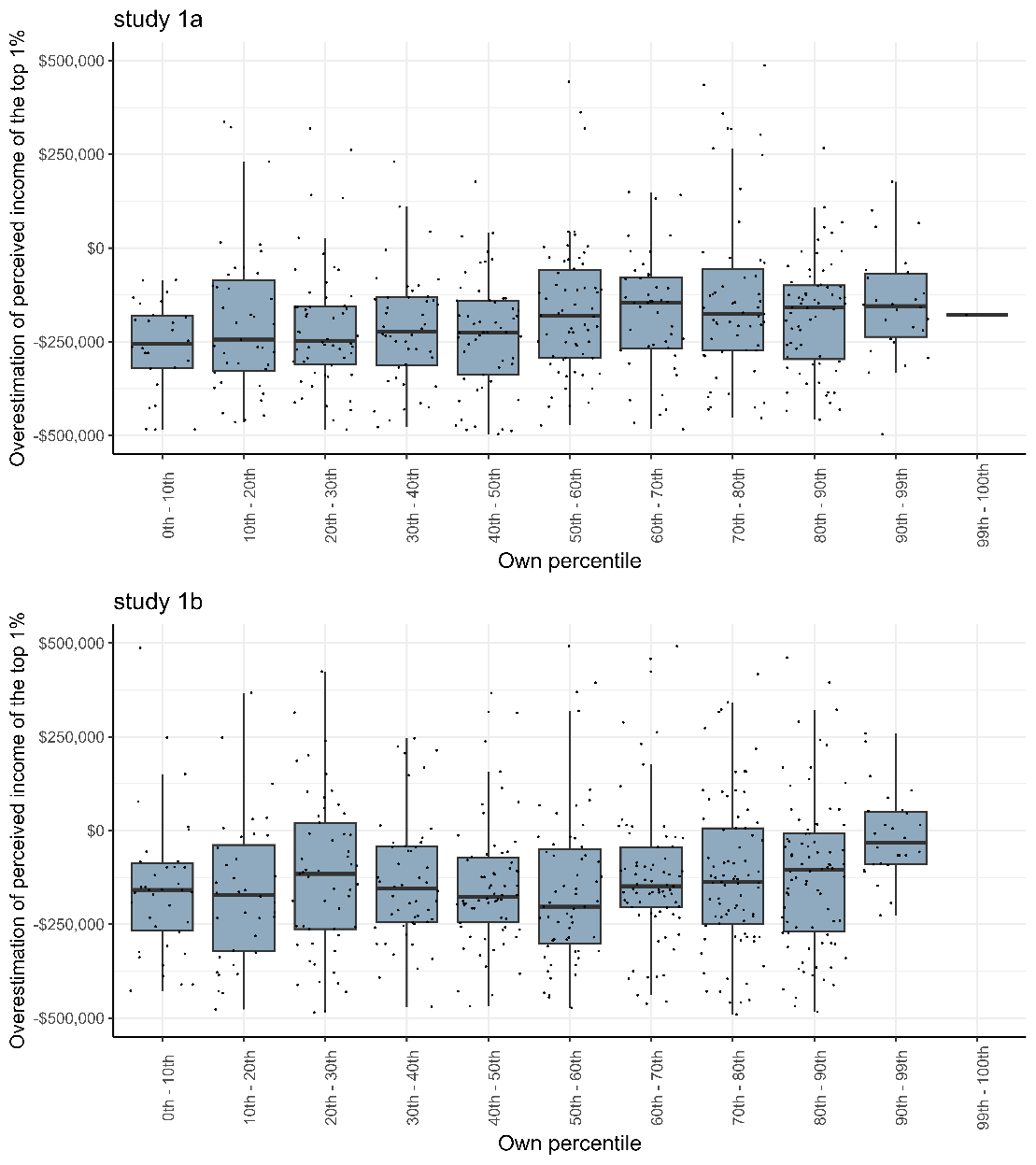


**Table S10.** Underestimation of income thresholds of the top 1% among participants with different annual income (Study 1a and 1b)

| percentile | n | Particpant Income | Median Underestimation | SD Underestimation | P | Z | Effect Size (r) |
| --- | --- | --- | --- | --- | --- | --- | --- |
| Study 1a |  |  |  |  |  |  |  |
| 99^th^ | 27 | 5000 | 267328 | 31875443 | < .001 | 3.892 | .749 |
| 99^th^ | 41 | 15000 | 194513 | 363913 | < .001 | 3.622 | .566 |
| 99^th^ | 63 | 25000 | 225130 | 329479 | < .001 | 4.861 | .612 |
| 99^th^ | 53 | 35000 | 224986 | 406834 | < .001 | 3.767 | .517 |
| 99^th^ | 61 | 45000 | 202512 | 127991368 | < .001 | 3.322 | .425 |
| 99^th^ | 62 | 55000 | 200408 | 12807438 | < .001 | 5.023 | .638 |
| 99^th^ | 34 | 65000 | 229096 | 378672 | <.001 | 4.334 | .743 |
| 99^th^ | 40 | 75000 | 167571 | 15809384 | <.001 | 3.629 | .574 |
| 99^th^ | 36 | 85000 | 223979 | 756598 | .004 | 2.906 | .484 |
| 99^th^ | 24 | 95000 | 122021 | 1041529 | .056 | 1.914 | .391 |
| 99^th^ | 51 | 112500 | 124634 | 140398912 | .18 | 1.34 | .188 |
| 99^th^ | 34 | 137500 | 278498 | 1848459 | <.001 | 3.445 | .591 |
| 99^th^ | 36 | 175000 | 238998 | 1666669950 | .001 | 3.236 | .539 |
| 99^th^ | 31 | 250000 | 189509 | 1796004131 | .063 | 1.862 | .334 |
| Study 1b |  |  |  |  |  |  |  |
| 99^th^ | 48 | 7500 | 158339 | 1412822 | .001 | 3.200 | .462 |
| 99^th^ | 48 | 20000 | 132758 | 846197 | .095 | 1.672 | .241 |
| 99^th^ | 77 | 30000 | 105023 | 1250621 | .014 | 2.455 | .280 |
| 99^th^ | 100 | 42500 | 137329 | 99976409 | .010 | 2.568 | .257 |
| 99^th^ | 135 | 62500 | 131645 | 2171224 | .003 | 2.974 | .256 |
| 99^th^ | 111 | 87500 | 154335 | 2163622 | .001 | 3.260 | .309 |
| 99^th^ | 105 | 125000 | 119120 | 812791 | .026 | 2.233 | .218 |
| 99^th^ | 47 | 175000 | 109457 | 818670 | .018 | 2.370 | .346 |
| 99^th^ | 41 | 250000 | 102494 | 813573 | .035 | 2.106 | .329 |

**Political Preferences do not Moderate Effects (Study 1a and 1b)**

We also tested whether political preferences moderated the degree to which citizens underestimated the income of the top 1 %. In Study 1a and Study 1b, we measured political preferences using the merged responses to the following questions, “How would you describe your political outlook with regard to social issues?” and "How would you describe your political outlook with regard to economic issues?”. Participants were asked to express their attitudes on a 7-point Likert scale ranging from "Very Liberal" to "Very Conservative.” Analyses showed that political orientation did not significantly shape whether participants underestimated the income of the top 1% for Study 1a (*p* = .846) nor Study 1b (*p* = .790).

**Study 2a**

**Figure S5.** Screenshot from Study 2a, showing avatars of 100 individuals in a fictional society. Once participants clicked on one avatar, the income of one individual appeared on the screen for 1.5 seconds.


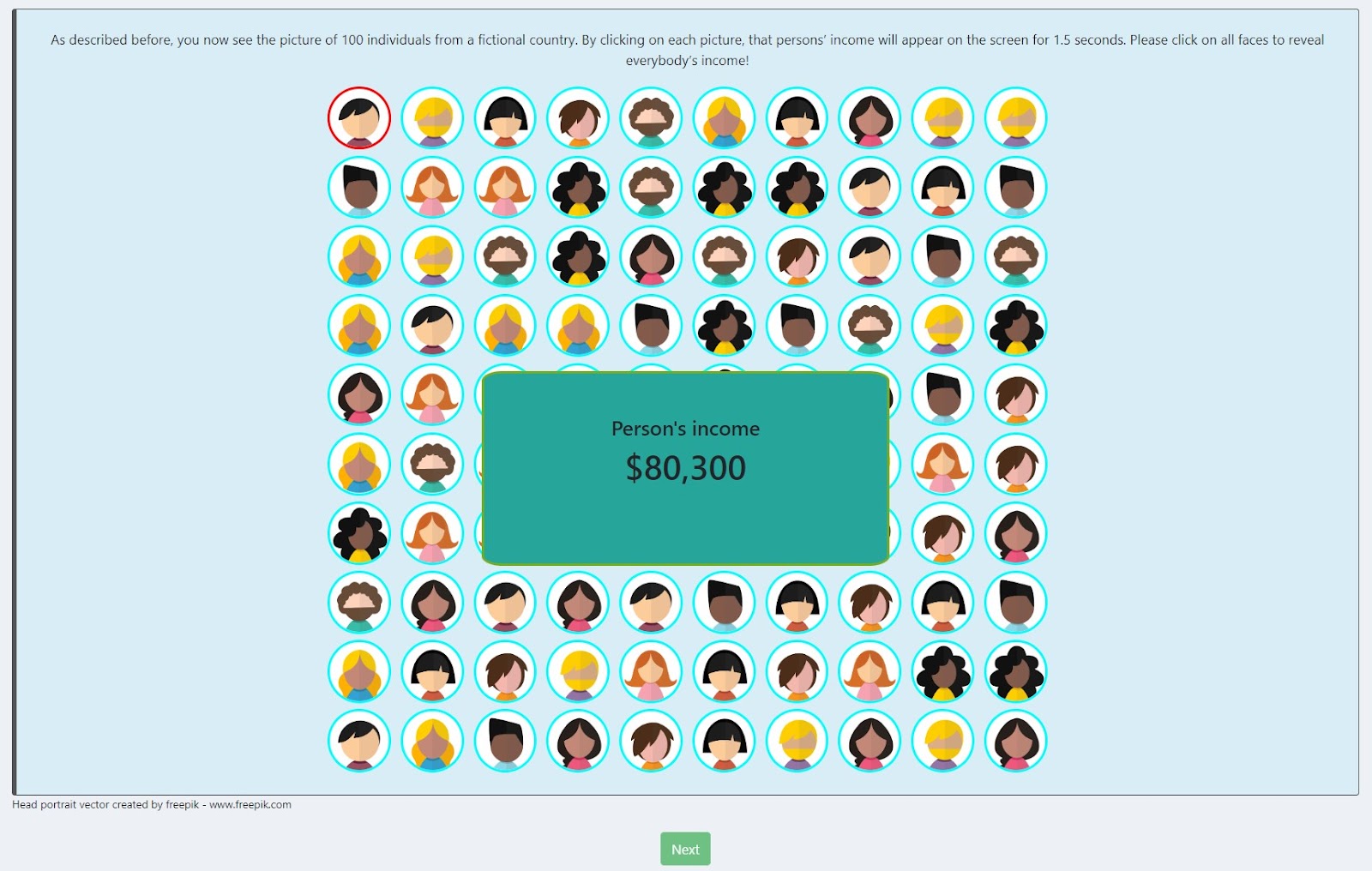


**Table S11.** Income brackets from [US Census Bureau, 2015](https://www.zotero.org/google-docs/?FinIwV), and the incomes presented in Study 2a and 2b.

| Income brackets | Proportion of population | Mean income | Proportion of population in the pooled income bracket | Mean Income in the pooled income brackets | Incomes presented in Study 3a and 3b (control and top 1% condition) |
| --- | --- | --- | --- | --- | --- |
| Under $5,000 | 3.67% | $1,08.00 | 12.6% | $7,826.80 | 6698, 9550, 12618, 1983, 8663, 6734, 10546, 11057, 1284, 8233, 12146, 6445, 5782 |
| $5,000 to $9,999 | 3.47% | $7,936.00 |  |  | 2302, 5276, 11404, 14636, 11831, 1838, 13350, 10199, 2694, 5822, 9537, 6897, 5967 |
| $10,000 to $14,999 | 5.43% | $12,317.00 |  |  |  |
| $15,000 to $19,999 | 5.44% | $17,338.00 | 16.1% | $22,099.47 | 23876, 22284, 18254, 26303, 29010, 21006, 19081, 19479, 27459, 19482, 23111, 21337, 29046, 23366, 15069, 15441 |
| $20,000 to $24,999 | 5.51% | $22,162.00 |  |  | 15385, 22287, 24381, 23449, 21183, 19450, 21389, 16789, 29219, 16242, 19697, 22541, 24478, 23786, 28254, 25062 |
| $25,000 to $29,999 | 5.11% | $27,101.00 |  |  |  |
| $30,000 to $34,999 | 5.00% | $32,058.00 | 14.1% | $36,806.91 | 31196, 42729, 33608, 40897, 34182, 30915, 37840, 32383, 35155, 41297, 41195, 31693, 40347, 41855 |
| $35,000 to $39,999 | 4.70% | $37,061.00 |  |  | 30661, 43228, 35659, 39818, 37121, 35392, 40505, 32551, 42425, 39716, 30053, 42509, 31500, 34159 |
| $40,000 to $44,999 | 4.36% | $41,979.00 |  |  |  |
| $45,000 to $49,999 | 4.06% | $47,207.00 | 11.53% | $51,796.50 | 50579, 51039, 47869, 45087, 54294, 56903, 59831, 48077, 49979, 59901, 46213 |
| $50,000 to $54,999 | 4.08% | $51,986.00 |  |  | 55882, 59612, 51638, 50918, 46106, 52649, 54923, 48462, 53889, 45497, 50196 |
| $55,000 to $59,999 | 3.39% | $57,065.00 |  |  |  |
| $60,000 to $64,999 | 3.59% | $62,016.00 | 9.6% | $66,738.64 | 69305, 63430, 60136, 65312, 69429, 74346, 66386, 60801, 72825, 65414 |
| $65,000 to $69,999 | 2.98% | $67,081.00 |  |  | 63465, 74368, 69628, 68066, 65272, 63206, 60416, 68810, 73782, 60365 |
| $70,000 to $74,999 | 3.00% | $72,05.00 |  |  |  |
| $75,000 to $79,999 | 2.80% | $77,023.00 | 7.5% | $81,635.05 | 88592, 89265, 84707, 79467, 79933, 75579, 77574, 77957 |
| $80,000 to $84,999 | 2.52% | $81,966.00 |  |  | 85414, 83673, 80293, 78033, 82641, 78985, 86255, 77783 |
| $85,000 to $89,999 | 2.21% | $87,101.00 |  |  |  |
| $90,000 to $94,999 | 2.14% | $92,033.00 | 6.2% | $97,041.08 | 93713, 97733, 101193, 90756, 100291, 98566 |
| $95,000 to $99,999 | 1.88% | $97,161.00 |  |  | 101424, 95808, 94807, 90499, 95614, 104094 |
| $100,000 to $104,999 | 2.15% | $101,921.00 |  |  |  |
| $105,000 to $109,999 | 1.66% | $107,187.00 | 4.5% | $111,731.02 | 118160, 111077, 105522, 112169 |
| $110,000 to $114,999 | 1.54% | $112,069.00 |  |  | 109393, 108552, 118314, 110666 |
| $115,000 to $119,999 | 1.30% | $117,133.00 |  |  |  |
| $120,000 to $124,999 | 1.50% | $122,127.00 | 3.9% | $126,682.72 | 129266, 132236, 124731, 120496 |
| $125,000 to $129,999 | 1.17% | $127,166.00 |  |  | 121463, 134799, 120305, 130162 |
| $130,000 to $134,999 | 1.21% | $131,863.00 |  |  |  |
| $135,000 to $139,999 | .98% | $137,284.00 | 2.8% | $141,926.72 | 135556, 147194, 143033 |
| $140,000 to $144,999 | 1.04% | $142,199.00 |  |  | 137534, 139035, 149209 |
| $145,000 to $149,999 | .82% | $147,13.00 |  |  |  |
| $150,000 to $154,999 | .92% | $151,94.00 | 2.3% | $156,555.85 | 150046, 163065 |
| $155,000 to $159,999 | .68% | $157,177.00 |  |  | 160847, 152265 |
| $160,000 to $164,999 | .70% | $162,019.00 |  |  |  |
| $165,000 to $169,999 | .63% | $167,101.00 | 1.7% | $171,737.22 | 176238, 167238 |
| $170,000 to $174,999 | .58% | $172,169.00 |  |  | 169003, 174473 |
| $175,000 to $179,999 | .49% | $177,187.00 |  |  |  |
| $180,000 to $184,999 | .50% | $182,055.00 | 1.3% | $186,78.63 | 186780 |
| $185,000 to $189,999 | .45% | $187,299.00 |  |  | 186781 |
| $190,000 to $194,999 | .39% | $192,241.00 |  |  |  |
| $195,000 to $199,999 | .35% | $197,211.00 | 3.0% | $217,54.78 | 212797, 195900, 243923 |
| $200,000 to $249,999 | 2.61% | $220,267.00 |  |  | 223850, 231223, 197552 |
| $250,000 and over | 3.02% | $402,476.00 | 3.0% | $402,476.00 | 312019, 299905, 595504 |
|  | | |  |  | 332876, 280215, 594336 |

**Table S12.** Medians and standard deviations of the underestimation, the results of the Clustered Wilcoxon tests and the corresponding Z statistics and effect sizes (Study 2a)

| Quintile | Median Underestimation | Mean Underestimation | SD Underestimation | *P* | *Z* | Effect size (r) |
| --- | --- | --- | --- | --- | --- | --- |
| top20 | 47279 | -17099463847 | 364426972796 | < .001 | 7.870 | .297 |
| second20 | -13007 | -7400513259 | 221006424389 | < .001 | -11.486 | .475 |
| middle20 | -6143 | -626977 | 18414254 | < .001 | -10.339 | .432 |
| fourth20 | -3240 | -6517 | 28211 | < .001 | -6.004 | .244 |
| bottom20 | 1478 | -3439 | 36191 | .841 | -0.200 | .008 |

**Figure S6.** Over- or underestimation of the income averages in each quintile (Study 2a).


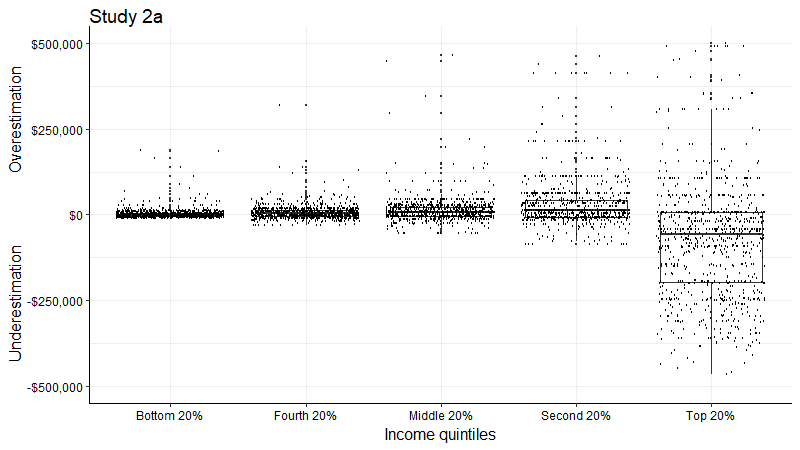


*Robustness tests (Study 2a)*

*Robustness analysis with different levels of income share of the top 1%*

To test whether our results hold for different income shares of the top 1%, we conducted Wilcoxon signed-rank tests to compare the differences between the objective and perceived income shares separately for each level of income share of the top 1% (35%, 40%, 45%, 50%) in the top 1% condition. The results showed significant difference between the objective and perceived difference for each level of the income share. Table S13 shows the test results of these analyses.

To check whether we see the same differential pattern for the top vs. the other quintiles, we also plotted the difference between the objective and perceived income shares in each quintile at each level of the top 1% income shares (Figure 4 in the main text) that suggest that individuals underestimate the income share of the top quintile at each level of income share for the top 1% but overestimate the other quintiles.

**Table S13.** The results of the Wilcoxon signed-rank tests at each income share of the top 1% (Study 2a)

| Income share of the top 1% | Median (objective) | Median  (perceived) | Mean (Perceived) | SD  (perceived) | | Effect size | *p* | Statistic |
| --- | --- | --- | --- | --- | --- | --- | --- | --- |
| 35 | 157042 | 20000 | 1467883 | 13758310 | .290 | | .002 | 2337 |
| 40 | 201416 | 40000 | 169964 | 420402 | .425 | | <.001 | 1475 |
| 45 | 253966 | 35000 | 26020812 | 278509666 | .447 | | <.001 | 1645 |
| 50 | 316866 | 100000 | 12635585 | 131074309 | .315 | | <.001 | 2087 |

*Robustness analysis after excluding participants with implausible answers (Study 2a)*

We excluded 38 participants who stated that the average income owned by one of the quintiles is 0 and who indicated that any richer quintile owns less than any of the poorer quintiles. The sample size after exclusions consisted of 417 individuals (48.2% female, M_age_= 36.59).

Again, we applied a Wilcoxon signed rank task to compare whether the objective or perceived difference was bigger between the conditions. The results revealed that the objective difference was significantly larger (*Mdn* = 201492, *Mean* = 230753) than the subjective difference (*Mdn* = 50000, *Mean* = 11141392, *p* < .001, *Z* = -6.319, *r* = .309), indicating that individuals were largely insensitive to the increased income of the top 1% in the top 1% condition compared to the control condition. In total, in 314 out of the 417 cases (75.3%), the respondents’ perception about the difference was lower than the objective values.

Finally, we collapsed across the two conditions, and calculated how much the perceived income average differed from the objective income average in each quintile. Using Clustered Wilcoxon tests clustered on participants, we tested separately in each quintile, whether this difference significantly differed from (Table S14, Figure S7).

**Figure S7.** Estimates of income averages in each quintile excluding participants with implausible answers (Study 2a)


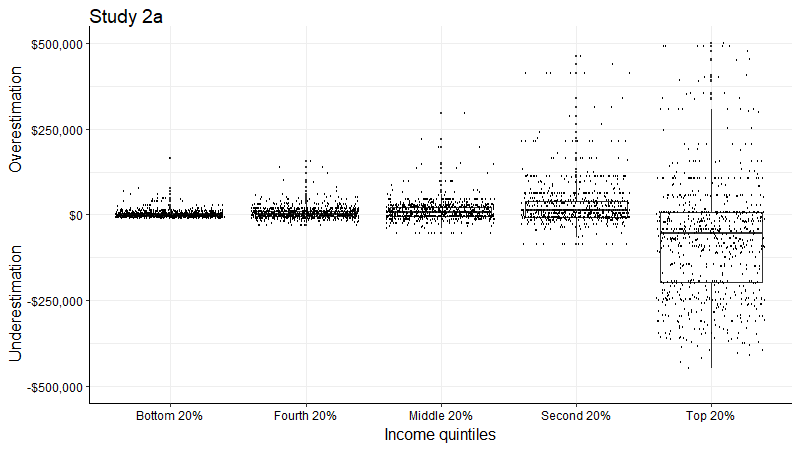


**Table S14.** Medians and standard deviations of the underestimation, the results of the Clustered Wilcoxon tests and the corresponding *Z* statistics and effect sizes (Study 2a)

| Quintile | Median Underesti-mation | Mean  Underesti-  mation | SD Underesti-mation | *P* | *Z* | Effect size *(r)* |
| --- | --- | --- | --- | --- | --- | --- |
| top20 | 44729 | -18657218038 | 380650463068 | <.001 | 7.287 | .285 |
| second20 | -13007 | -8073576057 | 230856553698 | <.001 | -11.259 | .492 |
| middle20 | -6143 | -678056 | 19235064 | <.001 | -9.817 | .430 |
| fourth20 | -1470 | -5107 | 18022 | <.001 | -5.126 | .218 |
| bottom20 | 1478 | -1620 | 11465 | .741 | 0.331 | .014 |

**Study 2b**

Similar to Study 2a, we collapsed the results of the treatment and control conditions and calculated how much the perceived income shares differed from the objective income averages in each quintile. Using Clustered Wilcoxon tests clustered on participants, we tested separately in each quintile, whether this difference significantly differed from 0. We repeated the same for both types of treatment separately (Table S15, Table S16, Figure S8).

**Table S15.** Medians and standard deviations of the underestimation of income averages for participants in top 1% treatment condition, the results of the Clustered Wilcoxon tests and the corresponding Z statistics and effect sizes in Study 2b. (Based on pooled top 1% treatment and control data.)

| Quintile | Median Underestimation | Mean Underestimation | SD Underestimation | *P* | *Z* | Effect size *(r)* |
| --- | --- | --- | --- | --- | --- | --- |
| top20 | 43245 | -334335 | 5282777 | <.001 | 5.146 | .292 |
| second20 | -13007 | -89688 | 958829 | <.001 | -7.967 | .484 |
| middle20 | -6143 | -18721 | 67554 | <.001 | -7.461 | .441 |
| fourth20 | -3470 | -6310 | 20031 | <.001 | -4.423 | .253 |
| bottom20 | 1478 | -491 | 7486 | .352 | 0.931 | .058 |

**Table S16.** Medians and standard deviations of the income average underestimation for participants in the top 10% treatment condition, the results of the Clustered Wilcoxon tests and the corresponding Z statistics and effect sizes in Study 2b. (Based on pooled top 1% treatment and control data.)

| Quintile | Median Underestimation | Mean Underestimation | SD Underestimation | *P* | *Z* | Effect size *(r)* |
| --- | --- | --- | --- | --- | --- | --- |
| top20 | -8254 | -2400786 | 45517364 | <.001 | -4.419 | .230 |
| second20 | -18058 | -102673 | 698797 | <.001 | -10.219 | .579 |
| middle20 | -11143 | -92502 | 1583215 | <.001 | -9.994 | .570 |
| fourth20 | -3470 | -70609 | 1357745 | <.001 | -5.444 | .294 |
| bottom20 | 1478 | -7065 | 83009 | .700 | 0.385 | .023 |

**Figure S8.** Estimates of the income thresholds in each quintile for the Top 1% and Top 10% treatment groups separately (Study 2b)


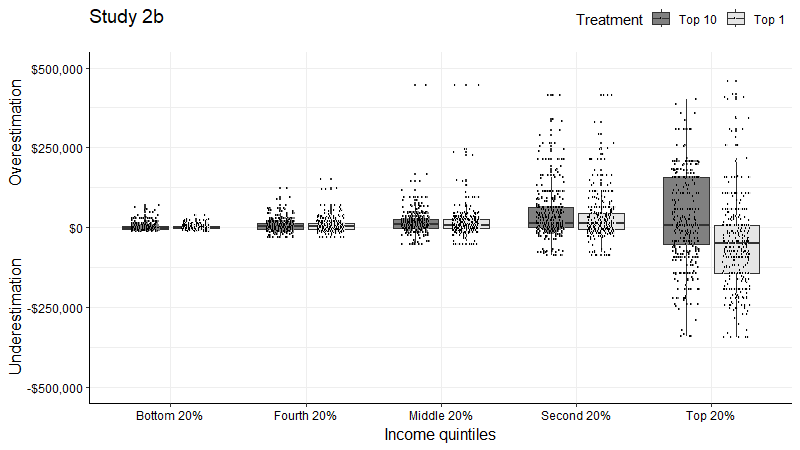


*Robustness tests (Study 2b)*

*Robustness analysis after excluding participants with implausible answers*

We excluded 48 participants who stated that the proportion of income (calculated from the provided averages) owned by one of the quintiles is 0 or 100 and who indicated that individuals in any richer quintile own less than individuals in any of the poorer quintiles. The sample size after exclusions consisted of 417 individuals (49.6% female, M_age_= 38.14).

Similar to the main analysis, we found that participants in the top 1% treatment condition underestimated the average income of the top 20% (*p* < .001, *Z* = -5.110, *r* = .360). We found the evidence in support of overestimation for participants in the top 10% treatment condition (*p* = .0227, *Z* = -3.523, *r* = .241). Critically, using a Manny-Whitney test, we found that the underestimation of the top 20% income bucket was larger for participants in the top 1% treatment condition in comparison to participants in the top 10% treatment condition (*p* < .001, *Z* = -6.115, *r* = .299).

Finally, we collapsed the two conditions, and calculated how much the perceived income averages differed from the objective income averages in each quintile. Using Clustered Wilcoxon tests clustered on participants, we tested separately in each quintile, whether this difference significantly differed from 0 (Table S17, TableS18, Figure S9).

**Table S17.** Medians and standard deviations of the underestimation in top 1% treatment, the results of the Clustered Wilcoxon tests and the corresponding *Z* statistics and effect sizes in Study 2b. (Pooled top 1% and control treatment.)

| Quintile | Median Underestimation | Mean Underestimation | SD Underestimation | *P* | *Z* | Effect size *(r)* |
| --- | --- | --- | --- | --- | --- | --- |
| top20 | 43245 | -348747 | 5504298 | < .001 | 4.557 | .273 |
| second20 | -13007 | -44243 | 158679 | < .001 | -8.115 | .522 |
| middle20 | -6143 | -16756 | 57037 | < .001 | -7.208 | .446 |
| fourth20 | -3355 | -6320 | 19966 | < .001 | -4.149 | .246 |
| bottom20 | 1478 | -346 | 7197 | .247 | 1.157 | .076 |

**Table S18.** Medians and standard deviations of the income share underestimation in top 10 % treatment, the results of the Clustered Wilcoxon tests and the corresponding Z statistics and effect sizes in Study 2b. (Pooled top 10% and control treatment.)

| Quintile | Median Underestimation | Mean Underestimation | SD Underestimation | *P* | *Z* | Effect size *(r)* |
| --- | --- | --- | --- | --- | --- | --- |
| top20 | -11721 | -2710306 | 48487816 | < .001 | -4.759 | .263 |
| second20 | -23007 | -99223 | 714962 | < .001 | -10.355 | .638 |
| middle20 | -13604 | -16483 | 33597 | < .001 | -9.965 | .615 |
| fourth20 | -4240 | -6217 | 16857 | < .001 | -5.345 | .304 |
| bottom20 | 1478 | -557 | 7913 | .177 | 1.350 | .085 |

**Figure S9.** Estimates of income thresholds in each quintile excluding participants with implausible answers (Study 2b)


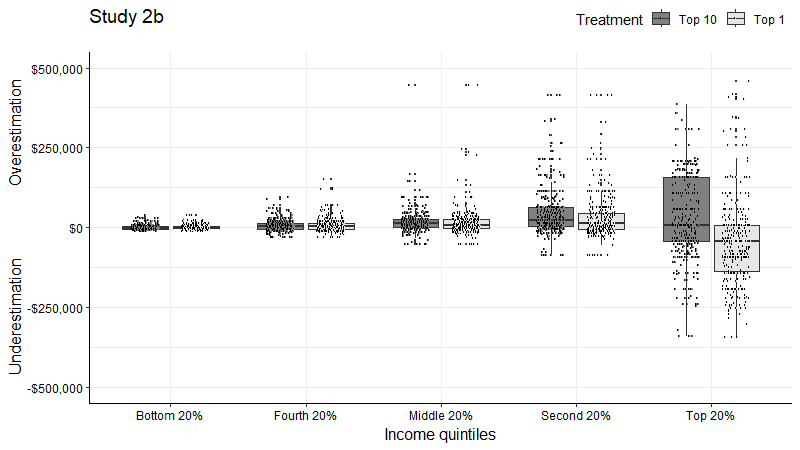


**Figure. S10.** Difference between objective and perceived income averages of the top quintile in the Top 1% and Top 10% treatment (Study 2b)


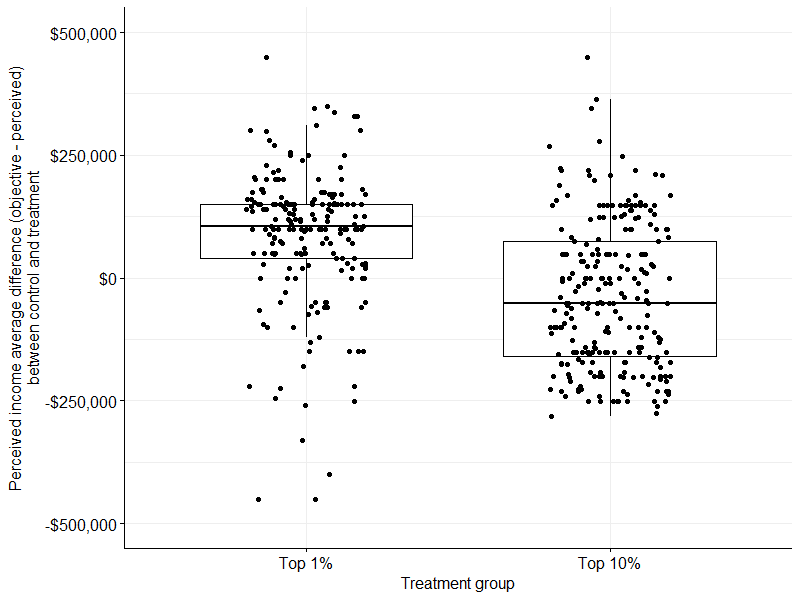


**Measuring affective reactions (Study 2b)**

For each individual, we calculated the proportion of incomes and the affective reactions for each neighboring income bracket, and for each neighboring bracket-pair, calculating the ratio of the “proportion of incomes" vs. the "proportion of affective reactions." To avoid the problem of dividing by 0, we added +1 to all affect scores. This ratio provides allows us to examine the change of affective reactions compared to the change of incomes. Visual inspection of Figures S11 and S12 suggests that there is a decreasing change in affective reactions compared to the change in incomes at the upper parts of the income distribution, in line with scope insensitivity.

**Figure S11.** The proportion of the incomes and the proportion of the affective reactions in each neighboring brackets (Study 2b)


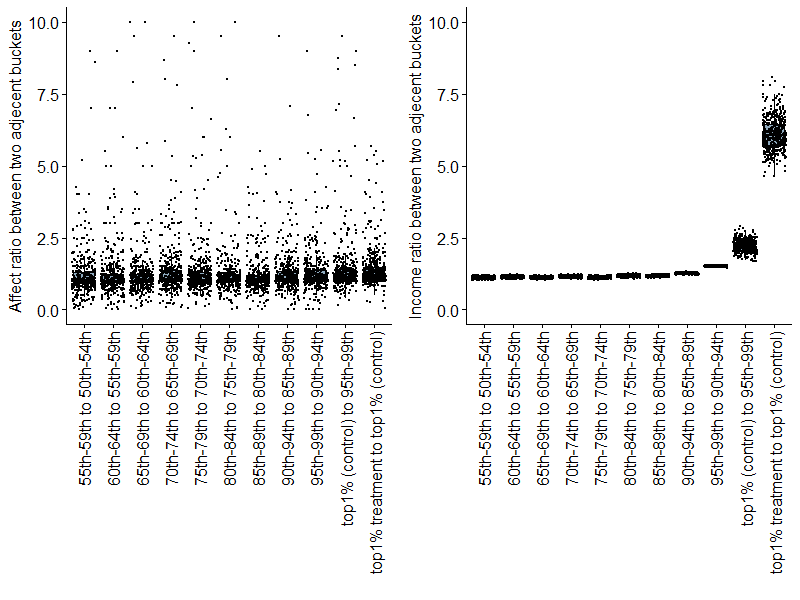


**Figure S12.** Ratio of the 'proportion of incomes' vs. the 'proportion of affective reactions' for each neighboring bracket pair (Study 2b)


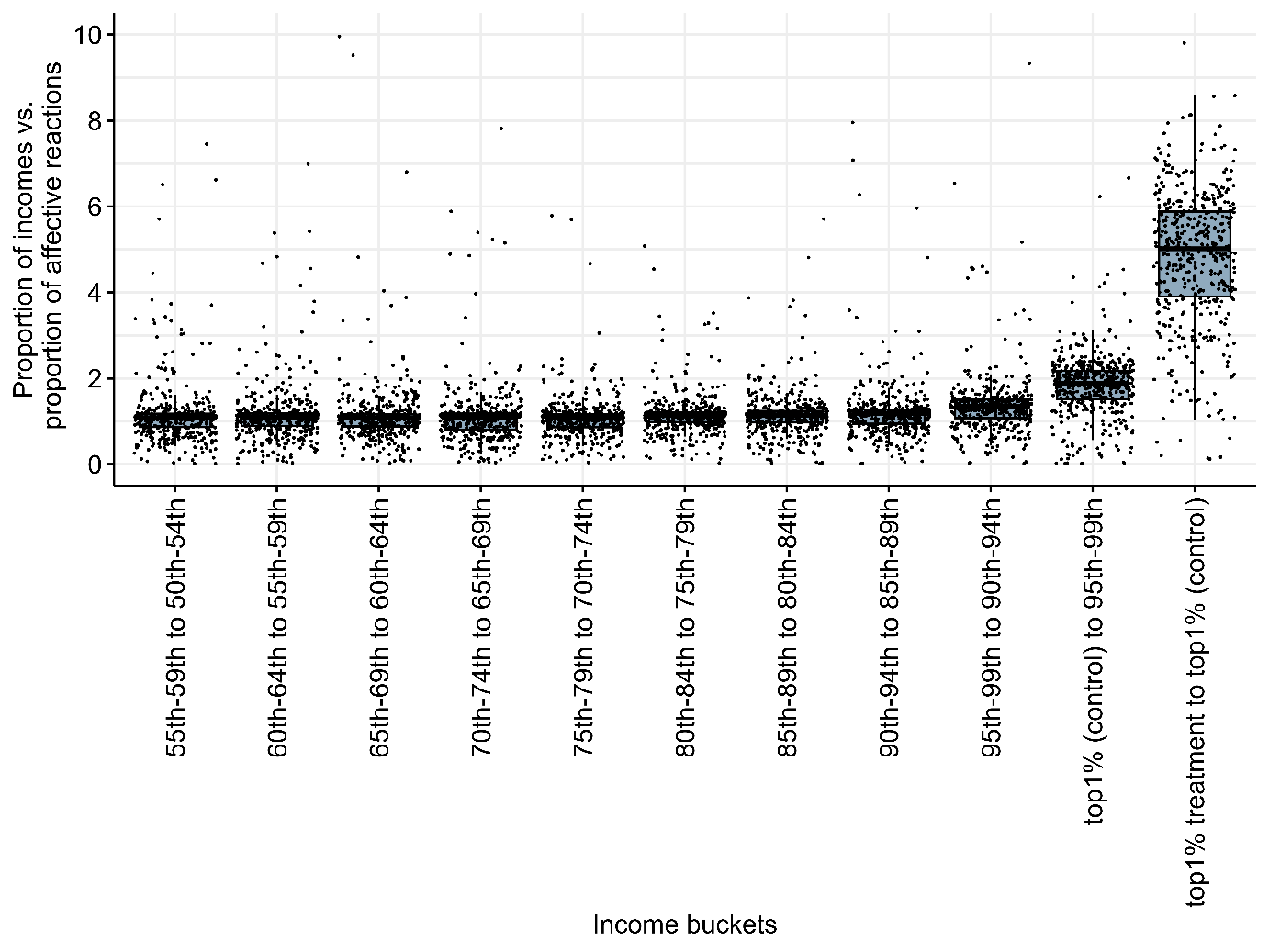


**Supplementary Study 1 (SS1)**

**Longitudinal Study in Representative Samples from 37 Countries**

In SS1, we aimed to provide broader generalizability for the unique underestimation of top incomes. In line with previous research, we obtained data on inequality perceptions from the International Social Survey Programme (ISSP). We only used the ISSP data where participants provided the exact estimates for incomes of the top 1%. The objective income data for each of these groups in each country for 1999 and for 2009 was downloaded from the World Inequality Database (<https://wid.world/>). We computed objective and perceived inequality estimates for countries where we could acquire both for a given year from the World Inequality Database, and from the ISSP, respectively. As a result, we used data from 19 and 40 countries from 1999 and 2009, respectively. Because we have separate datasets for East and West Germany, we conduct separate analyses on them.

Respondents in the ISSP survey were asked to estimate the incomes for different occupations. The exact wording was as follows: “How much do you think people in different jobs actually earn? **jobs listed**” in 1999 and “How much do you think a [**jobs**] earns?” in 2009. From the list of available occupations, we chose those for which we had data for both years (2009 and 1999) and which broadly fall into the top 1%, top 10% or bottom 50% based on their earnings and were used in previous research (S. Kiatpongsan, M. I. Norton, How much (more) should CEOs make? A universal desire for more equal pay. Perspect. Psychol. Sci. 9, 587–593 (2014)). As a result, we used participants’ estimates of ‘chairman of an international company’ as a proxy to estimate the perceived average income of the richest ~1% of the population. To estimate the perceived average income of the richest ~10% of the population, we used respondents’ estimates for ‘doctors in a general practice.’ To estimate the perceived average income of the poorest ~50% of the population, we averaged the estimates provided by each respondent for ‘unskilled workers’ and for ‘shop assistants.’ These professions are generally closely aligned with these income positions (see Supplementary Information Table S34). To approximate the perceived “top 1% income share / bottom 50% income share” inequality, we divided the top 1% income estimates with the bottom 50% income estimates multiplied by 50 for each respondent. To estimate the perceived “top 10% income share / bottom 50% income share” inequality, we divided the top 10% income estimates with the bottom 50% income estimates multiplied by 50 for each respondent. We subsequently calculated objective inequality measures as the ratio of the objective income share of the top 1% and of the bottom 50% of the individuals (*top 1% inequality*), and as the ratio of the objective income share of the top 10% (without the richest 1%) and the bottom 50% of individuals (*top 10% inequality*).

We excluded participants who provided unrealistic answers, that is, who estimated lower incomes for chairman of an international company than for unskilled workers or for shop assistants (631 and 2,632 participants were excluded from the 1999 and 2009 surveys, respectively). The final sample consisted of 23,288 participants in 1999 (M_age_ = 45.79, SD_age_ = 16.69, 51.73% female) from 19 countries, and 51,970 participants in 2009 (M_age_ = 46.88, SD_age_ = 17.24, 55.14% female) from 40 countries. We conducted a two-sided Wilcoxon rank test in each country to test whether participants under- or overestimated inequality at different income percentiles. Our analyses reveal that perceived top 1% inequality was significantly smaller than objective top 1% inequality in 17 out of 19 countries in 1999 (r_min,max_= [.002, .867], r_Mdn_ = .781) and in 32 out of 40 countries in 2009 (r_min,max_ = [.001, .866], r_Mdn_= .650) (see Figure 2). Similarly, perceived top 10% inequality was significantly smaller than objective top 10% inequality in 19 out of 19 countries in 1999 (r_min,max_ = [.349, .863], r_Mdn_ = .644) and 38 out of 40 countries in 2009 (r_min,max_ = [.110, .866], r_Mdn_ = .643). Finally, to test whether respondents underestimated inequality more among the top 1% than among the top 10%, we subtracted the perceived top 1% and top 10% inequality from the corresponding objective inequality values for each individual, and compared the degree of misperception between both level. In support of our hypothesis, a two-sided paired Wilcoxon rank test indicated that the underestimation of inequality was larger for the top 1% inequality than for the top 10% inequality in all countries in 1999, and in 36 out of 40 countries in 2009 **(**see Table S21, and Table S22 for further information.)

**Figure S13.** Accuracy of top 1% inequality perceptions, plotted separately by country and for survey years 1999 (left panel) and 2009 (right panel). The top 1% inequality was underestimated in almost all countries, but critically the underestimation of inequality was larger for the top 1% inequality than for the top 10% inequality in all countries in 1999, and in 36 out of 40 countries in 2009 (see Figure S14 for top 10% data). Data from East and West Germany are plotted separately, in line with the data collection of the ISSP^5^. (SS1)


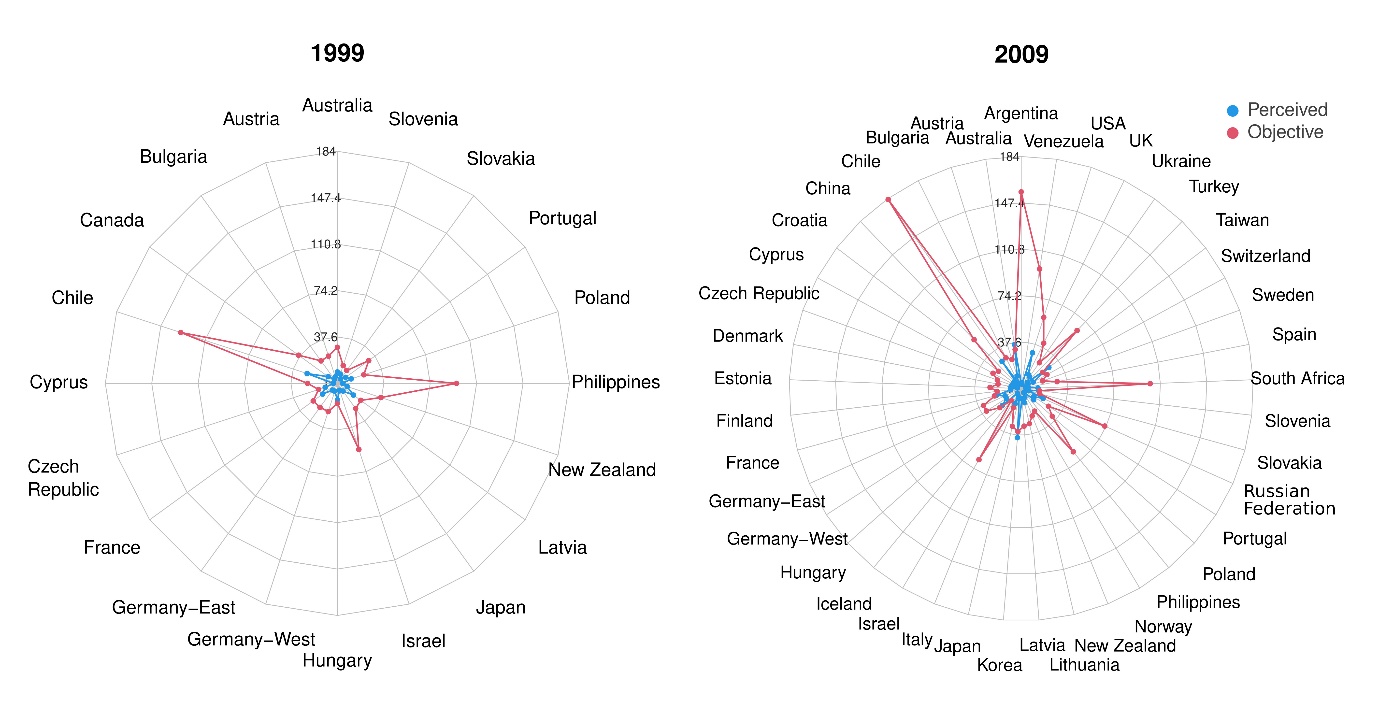


**Table S19.** Descriptives of participant samples for each country in 1999 (SS1)

| Country | *n* | % of Females | *M(age)* | *SD*(age) |
| --- | --- | --- | --- | --- |
| Australia | 1669 | 51.468 | 46.870 | 15.854 |
| Austria | 1014 | 58.580 | 49.831 | 16.655 |
| Bulgaria | 1021 | 51.910 | 48.583 | 17.230 |
| Canada | 959 | 35.975 | 40.264 | 14.792 |
| Chile | 1478 | 56.157 | 44.018 | 17.607 |
| Cyprus | 995 | 49.950 | 40.866 | 14.199 |
| Czech Republic | 1815 | 54.711 | 50.292 | 16.828 |
| France | 1873 | 41.431 | 48.978 | 14.961 |
| Germany-East | 511 | 51.468 | 48.121 | 17.458 |
| Germany-West | 917 | 51.690 | 48.135 | 17.044 |
| Hungary | 1184 | 56.926 | 48.518 | 17.831 |
| Israel | 1124 | 52.758 | 39.862 | 16.196 |
| Japan | 1324 | 52.115 | 45.810 | 17.596 |
| Latvia | 898 | 56.013 | 42.853 | 15.601 |
| New Zealand | 1105 | 50.498 | 47.245 | 16.141 |
| Philippines | 1113 | 50.135 | 39.324 | 15.041 |
| Poland | 1085 | 57.880 | 46.987 | 17.682 |
| Portugal | 1134 | 53.527 | 46.061 | 15.817 |
| Slovakia | 1078 | 52.690 | 42.965 | 15.981 |
| Slovenia | 991 | 51.261 | 45.479 | 16.292 |
| **Total** | **23288** | **51.734** | **45.792** | **16.692** |

**Table S20.** Descriptives of participants samples for each country in in 2009 (SS1)

| Country | *n* | % of Females | *M(age)* | *SD*(age) |
| --- | --- | --- | --- | --- |
| Argentina | 1067 | 50.234 | 46.514 | 17.544 |
| Australia | 1516 | 55.805 | 52.480 | 16.752 |
| Austria | 1018 | 52.554 | 45.055 | 17.086 |
| Bulgaria | 990 | 57.980 | 49.057 | 17.241 |
| Chile | 1494 | 58.032 | 46.573 | 17.636 |
| China | 1648 | 54.733 | 43.447 | 14.500 |
| Croatia | 1201 | 56.953 | 45.708 | 17.562 |
| Cyprus | 1000 | 50.600 | 42.624 | 15.415 |
| Czech Republic | 1195 | 55.146 | 46.883 | 16.773 |
| Denmark | 1435 | 53.519 | 49.981 | 17.124 |
| Estonia | 980 | 64.592 | 50.998 | 18.727 |
| Finland | 869 | 54.200 | 47.649 | 16.497 |
| France | 2802 | 51.499 | 55.073 | 15.678 |
| Germany-East | 436 | 48.165 | 51.348 | 17.800 |
| Germany-West | 952 | 50.420 | 48.810 | 17.884 |
| Hungary | 1006 | 54.473 | 46.187 | 15.922 |
| Iceland | 940 | 52.340 | 46.101 | 17.307 |
| Israel | 1166 | 52.830 | 43.442 | 17.552 |
| Italy | 1071 | 53.782 | 48.323 | 17.064 |
| Japan | 1294 | 53.400 | 49.227 | 17.571 |
| Korea | 1591 | 51.791 | 43.490 | 15.258 |
| Latvia | 1037 | 61.427 | 44.429 | 16.891 |
| Lithuania | 994 | 66.298 | 51.463 | 18.472 |
| New Zealand | 929 | 55.436 | 50.542 | 16.967 |
| Norway | 1179 | 51.739 | 47.722 | 15.260 |
| Philippines | 1117 | 49.955 | 42.614 | 16.010 |
| Poland | 1252 | 54.393 | 45.895 | 17.106 |
| Portugal | 991 | 59.334 | 49.395 | 18.097 |
| Russian Federation | 1339 | 64.675 | 47.687 | 18.221 |
| Slovakia | 1154 | 60.139 | 46.278 | 16.317 |
| Slovenia | 1065 | 54.648 | 46.655 | 17.821 |
| South Africa | 3225 | 59.814 | 39.400 | 15.703 |
| Spain | 1206 | 51.327 | 47.085 | 17.815 |
| Sweden | 1114 | 52.513 | 48.483 | 16.359 |
| Switzerland | 1227 | 55.338 | 50.082 | 17.669 |
| Taiwan | 2025 | 48.148 | 44.815 | 16.596 |
| Turkey | 1539 | 52.632 | 41.170 | 15.453 |
| Ukraine | 1567 | 65.412 | 49.205 | 17.538 |
| United Kingdom | 936 | 55.662 | 50.120 | 17.207 |
| USA | 1551 | 54.610 | 49.540 | 17.101 |
| Venezuela | 852 | 48.709 | 36.972 | 15.095 |
| **Total** | **51970** | **55.140** | **46.884** | **17.236** |

**Table S21.** Underestimation of the Top 1% / Bottom 50% Income Share Ratio in 1999 (SS1)

| Country | *Median*  Perceived Ratio | *SD*  Perceived  Ratio | Objective  Ratio | *P* | Wilcoxon test Statistic | | Effect size *(r)* |
| --- | --- | --- | --- | --- | --- | --- | --- |
| Australia | 10 | 5.05 | 29.459 | < .001 | 331 | .866 | |
| Austria | 6.452 | 7.718 | 23.579 | < .001 | 9395 | .821 | |
| Bulgaria | 5 | 9.475 | 22.941 | < .001 | 2222 | .850 | |
| Canada | 10 | 71.578 | 38.813 | < .001 | 47720 | .668 | |
| Chile | 26.087 | 210.624 | 130.906 | < .001 | 30130 | .739 | |
| Cyprus | 4.211 | 3.009 | 24.515 | < .001 | 6 | .866 | |
| Czech Republic | 11.111 | 13.822 | 16.573 | < .001 | 479429 | .234 | |
| France | 15.385 | 25.004 | 24.527 | < .001 | 505939 | .247 | |
| Germany-East | 7.5 | 6.753 | 24.342 | < .001 | 683 | .85 | |
| Germany-West | 7.5 | 6.463 | 24.342 | < .001 | 1516 | .852 | |
| Hungary | 14 | 32.863 | 16.768 | .963 | 188661 | .002 | |
| Israel | 6.667 | 7.042 | 55.792 | < .001 | 0 | .866 | |
| Japan | 8.485 | 23.473 | 25.549 | < .001 | 26880 | .637 | |
| Latvia | 16.667 | 71.582 | 23.774 | .248 | 163244 | .04 | |
| New Zealand | 9.091 | 30.668 | 37.091 | < .001 | 20405 | .797 | |
| Philippines | 5.298 | 487.198 | 94.962 | < .001 | 4951 | .848 | |
| Poland | 12.5 | 54.009 | 22.911 | < .001 | 108435 | .341 | |
| Portugal | 9.091 | 15.115 | 31.582 | < .001 | 27602 | .764 | |
| Slovakia | 5.333 | 2.382 | 13.570 | < .001 | 0 | .867 | |
| Slovenia | 8.696 | 13.073 | 15.823 | < .001 | 67749 | .588 | |

**Table S22.** Underestimation of the Top 1% / Bottom 50% Income Share Ratio in 2009 (SS1)

| Country | *Median*  Perceived Ratio | *SD*  Perceived  Ratio | Objective  Ratio | *P* | Wilcoxon test Statistic | Effect size *(r)* |
| --- | --- | --- | --- | --- | --- | --- |
| Argentina | 5.714 | 18.284 | 156.954 | < .001 | 83 | .866 |
| Australia* | 36.364 | 271361.374 | 32.385 | < .001 | 634756 | .325 |
| Austria | 11.538 | 47.426 | 25.269 | < .001 | 146982 | .318 |
| Bulgaria | 5.714 | 6.132 | 28.264 | < .001 | 173 | .864 |
| Chile | 27.429 | 5293.753 | 184.298 | < .001 | 56098 | .757 |
| China | 4.651 | 451.478 | 54.880 | < .001 | 41522 | .751 |
| Croatia | 8.571 | 11.046 | 23.686 | < .001 | 56001 | .722 |
| Cyprus | 6.250 | 2.641 | 26.426 | < .001 | 1 | .866 |
| Czech Republic | 8.889 | 12.838 | 21.090 | < .001 | 60300 | .704 |
| Denmark | 4.000 | 19.639 | 19.672 | < .001 | 68264 | .719 |
| Estonia | 9.499 | 24.133 | 25.714 | < .001 | 19784 | .785 |
| Finland | 9.375 | 35.443 | 20.193 | < .001 | 73568 | .465 |
| France* | 21.429 | 11539.206 | 22.874 | < .001 | 1666652 | .207 |
| Germany-East | 15.000 | 492.346 | 33.742 | < .001 | 29697 | .174 |
| Germany-West | 15.000 | 359.285 | 33.742 | .004 | 143763 | .100 |
| Hungary | 21.053 | 22.056 | 23.691 | .985 | 162483 | .001 |
| Iceland | 5.769 | 9.019 | 13.417 | < .001 | 31419 | .725 |
| Israel | 6.429 | 12.030 | 66.505 | < .001 | 2535 | .856 |
| Italy | 14.000 | 334.399 | 17.185 | .476 | 223105 | .023 |
| Japan | 8.333 | 61.632 | 31.686 | < .001 | 27161 | .650 |
| Korea* | 40.000 | 41669.621 | 35.099 | < .001 | 826003 | .321 |
| Latvia | 8.824 | 30.535 | 30.843 | < .001 | 41845 | .650 |
| Lithuania | 12.500 | 41.259 | 29.525 | < .001 | 47800 | .634 |
| New Zealand | 8.571 | 29.481 | 24.291 | < .001 | 65465 | .561 |
| Norway | 4.762 | 22.246 | 21.765 | < .001 | 42957 | .750 |
| Philippines | 4.615 | 1905.497 | 65.919 | < .001 | 13616 | .806 |
| Poland | 14.286 | 51.719 | 34.011 | < .001 | 91571 | .481 |
| Portugal | 12.632 | 35.852 | 26.744 | < .001 | 45866 | .429 |
| Russian Federation | 20.000 | 138.223 | 73.619 | < .001 | 109791 | .560 |
| Slovakia | 7.143 | 55.025 | 16.254 | < .001 | 67595 | .664 |
| Slovenia | 15.000 | 10.866 | 15.567 | .932 | 184013 | .003 |
| South Africa | 14.286 | 78.073 | 103.337 | < .001 | 148512 | .807 |
| Spain | 6.667 | 11.949 | 30.005 | < .001 | 14646 | .793 |
| Sweden | 4.651 | 44.363 | 19.025 | < .001 | 58761 | .670 |
| Switzerland | 11.765 | 269.560 | 24.232 | < .001 | 179867 | .311 |
| Taiwan* | 28.571 | 311.948 | 22.277 | < .001 | 1008459 | .412 |
| Turkey | 8.000 | 15.095 | 64.887 | < .001 | 6113 | .852 |
| Ukraine | 12.500 | 82.483 | 26.087 | < .001 | 55747 | .390 |
| United Kingdom | 13.636 | 5027.263 | 41.193 | < .001 | 65218 | .527 |
| USA | 30.769 | 1000.327 | 60.231 | .082 | 472112 | .046 |
| Venezuela | 4.558 | 14.062 | 96.956 | < .001 | 818 | .862 |

Note. For countries marked with *, perceived inequality is larger than objective inequality.

**Table S23.** Underestimation of the Top 10% / Bottom 50% Income Share Ratio in 1999 (SS1)

| Country | Median  Perceived Ratio | SD Perceived  Ratio | Objective  Ratio | *P* | Wilcoxon test Statistic | | Effect size *(r)* |
| --- | --- | --- | --- | --- | --- | --- | --- |
| Australia | 4.000 | 2.482 | 6.586 | < .001 | 170676 | .626 | |
| Austria | 4.000 | 4.727 | 5.459 | < .001 | 111306 | .349 | |
| Bulgaria | 2.000 | 1.064 | 6.098 | < .001 | 1643 | .856 | |
| Canada | 4.500 | 4.358 | 8.378 | < .001 | 43294 | .689 | |
| Chile | 5.517 | 19.224 | 17.792 | < .001 | 43804 | .722 | |
| Cyprus | 4.359 | 3.323 | 6.902 | < .001 | 70626 | .619 | |
| Czech Republic | 2.667 | 1.566 | 3.780 | < .001 | 225726 | .567 | |
| France | 3.846 | 3.388 | 5.776 | < .001 | 223203 | .620 | |
| Germany-East | 3.500 | 2.221 | 5.507 | < .001 | 12309 | .644 | |
| Germany-West | 3.750 | 2.937 | 5.507 | < .001 | 58114 | .508 | |
| Hungary | 2.286 | 2.733 | 4.256 | < .001 | 50461 | .631 | |
| Israel | 2.364 | 1.822 | 12.261 | < .001 | 3085 | .857 | |
| Japan | 5.000 | 8.598 | 8.490 | < .001 | 44297 | .509 | |
| Latvia | 1.556 | 0.900 | 7.517 | < .001 | 770 | .863 | |
| New Zealand | 4.103 | 5.063 | 7.121 | < .001 | 55370 | .680 | |
| Philippines | 3.611 | 12.587 | 14.026 | < .001 | 34917 | .743 | |
| Poland | 2.143 | 3.353 | 5.108 | < .001 | 22553 | .758 | |
| Portugal | 4.000 | 7.133 | 8.029 | < .001 | 63468 | .639 | |
| Slovakia | 3.333 | 1.561 | 4.328 | < .001 | 140075 | .433 | |
| Slovenia | 2.909 | 2.797 | 5.296 | < .001 | 15615 | .803 | |

**Table S24.** Underestimation of the Top 10% / Bottom 50% Income Share Ratio in 2009 (SS1)

| Country | *Median*  Perceived Ratio | *SD*  Perceived  Ratio | Objective  Ratio | *P* | Wilcoxon test Statistic | Effect size *(r)* |
| --- | --- | --- | --- | --- | --- | --- |
| Argentina | 2.286 | 4.933 | 12.204 | < .001 | 2163 | .851 |
| Australia | 5.000 | 3247.571 | 6.360 | < .001 | 388178 | .146 |
| Austria | 4.000 | 4.379 | 6.077 | < .001 | 121331 | .424 |
| Bulgaria | 3.636 | 3.011 | 6.520 | < .001 | 17938 | .610 |
| Chile | 7.273 | 1132.801 | 22.209 | < .001 | 114069 | .659 |
| China | 1.667 | 2.857 | 10.651 | < .001 | 6331 | .850 |
| Croatia | 3.000 | 1.277 | 7.386 | < .001 | 3185 | .858 |
| Cyprus* | 9.412 | 4.711 | 7.352 | < .001 | 385379 | .554 |
| Czech Republic | 3.333 | 2.562 | 3.943 | < .001 | 242641 | .206 |
| Denmark | 3.261 | 7.924 | 4.494 | < .001 | 187419 | .471 |
| Estonia | 2.857 | 1.648 | 7.320 | < .001 | 3311 | .853 |
| Finland | 2.632 | 2.331 | 5.944 | < .001 | 17191 | .773 |
| France | 4.000 | 5.644 | 5.668 | < .001 | 854404 | .331 |
| Germany-East | 4.000 | 3.152 | 7.023 | < .001 | 8599 | .669 |
| Germany-West | 3.846 | 35.937 | 7.023 | < .001 | 56282 | .566 |
| Hungary | 2.727 | 2.523 | 5.239 | < .001 | 31898 | .707 |
| Iceland | 3.333 | 2.148 | 4.802 | < .001 | 63932 | .579 |
| Israel | 2.182 | 1.478 | 13.566 | < .001 | 5 | .866 |
| Italy | 3.500 | 6.063 | 5.769 | < .001 | 61168 | .643 |
| Japan | 5.714 | 17.882 | 9.737 | < .001 | 44111 | .541 |
| Korea | 6.667 | 18.838 | 9.909 | < .001 | 325460 | .408 |
| Latvia | 2.400 | 1.934 | 8.009 | < .001 | 4305 | .845 |
| Lithuania | 2.500 | 2.427 | 7.888 | < .001 | 5474 | .840 |
| New Zealand | 4.000 | 3.494 | 5.857 | < .001 | 82026 | .492 |
| Norway | 2.909 | 15.133 | 4.431 | < .001 | 95538 | .608 |
| Philippines | 4.444 | 1906.301 | 13.264 | < .001 | 45354 | .669 |
| Poland | 3.429 | 3.966 | 6.362 | < .001 | 68341 | .571 |
| Portugal | 5.000 | 9.832 | 8.292 | < .001 | 24686 | .646 |
| Russian Federation | 1.667 | 0.918 | 10.854 | < .001 | 0 | .866 |
| Slovakia | 2.501 | 1.663 | 4.530 | < .001 | 59643 | .689 |
| Slovenia | 4.706 | 4.179 | 5.531 | .001 | 154566 | .110 |
| South Africa | 10.000 | 41.134 | 25.158 | < .001 | 674464 | .604 |
| Spain | 2.667 | 2.323 | 6.376 | < .001 | 10165 | .825 |
| Sweden | 2.250 | 1.383 | 4.730 | < .001 | 7814 | .841 |
| Switzerland | 3.333 | 4.026 | 5.165 | < .001 | 98762 | .567 |
| Taiwan* | 7.273 | 185.345 | 6.163 | < .001 | 1201504 | .327 |
| Turkey | 3.636 | 3.131 | 12.358 | < .001 | 7383 | .852 |
| Ukraine | 1.500 | 0.750 | 6.566 | < .001 | 854 | .862 |
| United Kingdom | 5.161 | 241.701 | 7.456 | < .001 | 77422 | .473 |
| USA | 6.000 | 273.280 | 10.355 | < .001 | 235645 | .459 |
| Venezuela | 2.143 | 2.581 | 15.760 | < .001 | 1690 | .858 |

Note. For countries marked with *, perceived inequality is larger than objective inequality.

**Table S25.** Underestimation of the Top 1% / Bottom 50% Income Share Ratio in 1999 (Winsorized 90%) (SS1)

| Country | *Median*  Perceived Ratio | *SD*  Perceived  Ratio | Objective  Ratio | *P* | Wilcoxon test Statistic | | Effect size *(r)* |
| --- | --- | --- | --- | --- | --- | --- | --- |
| Australia | 10.000 | 3.857 | 29.459 | < .001 | 0 | .867 | |
| Austria | 6.452 | 6.061 | 23.579 | < .001 | 1401 | .860 | |
| Bulgaria | 5.000 | 3.033 | 22.941 | < .001 | 0 | .866 | |
| Canada | 10.000 | 17.902 | 38.813 | < .001 | 36953 | .713 | |
| Chile | 26.087 | 45.883 | 130.906 | < .001 | 6251 | .840 | |
| Cyprus | 4.211 | 2.387 | 24.515 | < .001 | 0 | .866 | |
| Czech Republic | 11.111 | 11.038 | 16.573 | < .001 | 481039 | .232 | |
| France | 15.385 | 20.019 | 24.527 | < .001 | 506377 | .246 | |
| Germany-East | 7.500 | 5.862 | 24.342 | < .001 | 0 | .867 | |
| Germany-West | 7.500 | 5.542 | 24.342 | < .001 | 0 | .866 | |
| Hungary | 14.000 | 18.294 | 16.768 | .995 | 188956 | .000 | |
| Israel | 6.667 | 5.670 | 55.792 | < .001 | 0 | .866 | |
| Japan | 8.485 | 12.045 | 25.549 | < .001 | 25024 | .653 | |
| Latvia | 16.667 | 29.396 | 23.774 | .256 | 163385 | .039 | |
| New Zealand | 9.091 | 10.778 | 37.091 | < .001 | 3839 | .853 | |
| Philippines | 5.298 | 8.765 | 94.962 | < .001 | 0 | .866 | |
| Poland | 12.500 | 19.856 | 22.911 | < .001 | 108473 | .341 | |
| Portugal | 9.091 | 9.292 | 31.582 | < .001 | 4525 | .850 | |
| Slovakia | 5.333 | 2.274 | 13.570 | < .001 | 0 | .867 | |
| Slovenia | 8.696 | 6.229 | 15.823 | < .001 | 57385 | .631 | |

**Table S26.** Underestimation of the Top 1% / Bottom 50% Income Share Ratio in 2009 (Winsorized 90%) (SS1)

| Country | *Median*  Perceived Ratio | *SD*  Perceived  Ratio | Objective  Ratio | *P* | Wilcoxon test Statistic | Effect size *(r)* |
| --- | --- | --- | --- | --- | --- | --- |
| Argentina | 5.714 | 5.392 | 156.954 | < .001 | 0 | .866 |
| Australia* | 36.364 | 66.161 | 32.385 | < .001 | 635130 | .326 |
| Austria | 11.538 | 26.331 | 25.269 | < .001 | 147070 | .317 |
| Bulgaria | 5.714 | 5.054 | 28.264 | < .001 | 0 | .867 |
| Chile | 27.429 | 52.502 | 184.298 | < .001 | 3924 | .859 |
| China | 4.651 | 17.615 | 54.880 | < .001 | 4208 | .855 |
| Croatia | 8.571 | 7.683 | 23.686 | < .001 | 17831 | .820 |
| Cyprus | 6.250 | 2.208 | 26.426 | < .001 | 0 | .866 |
| Czech Republic | 8.889 | 7.167 | 21.090 | < .001 | 25395 | .798 |
| Denmark | 4.000 | 7.060 | 19.672 | < .001 | 14224 | .836 |
| Estonia | 9.499 | 6.314 | 25.714 | < .001 | 0 | .866 |
| Finland | 9.375 | 12.686 | 20.193 | < .001 | 73598 | .465 |
| France* | 21.429 | 62.210 | 22.874 | < .001 | 1667342 | .208 |
| Germany-East | 15.000 | 128.073 | 33.742 | < .001 | 29697 | .174 |
| Germany-West | 15.000 | 149.089 | 33.742 | .004 | 143794 | .100 |
| Hungary | 21.053 | 17.662 | 23.691 | .969 | 162866 | .001 |
| Iceland | 5.769 | 3.867 | 13.417 | < .001 | 8722 | .827 |
| Israel | 6.429 | 5.124 | 66.505 | < .001 | 0 | .866 |
| Italy | 14.000 | 31.293 | 17.185 | .490 | 223294 | .022 |
| Japan | 8.333 | 16.332 | 31.686 | < .001 | 27161 | .650 |
| Korea | 40.000 | 519.997 | 35.099 | < .001 | 828412 | .325 |
| Latvia | 8.824 | 14.489 | 30.843 | < .001 | 30425 | .709 |
| Lithuania | 12.500 | 13.093 | 29.525 | < .001 | 35339 | .694 |
| New Zealand | 8.571 | 14.623 | 24.291 | < .001 | 65506 | .561 |
| Norway | 4.762 | 6.377 | 21.765 | < .001 | 4831 | .853 |
| Philippines | 4.615 | 9.067 | 65.919 | < .001 | 0 | .866 |
| Poland | 14.286 | 25.237 | 34.011 | < .001 | 91652 | .481 |
| Portugal | 12.632 | 16.411 | 26.744 | < .001 | 46176 | .426 |
| Russian Federation | 20.000 | 45.235 | 73.619 | < .001 | 109791 | .560 |
| Slovakia | 7.143 | 6.192 | 16.254 | < .001 | 42493 | .739 |
| Slovenia | 15.000 | 9.714 | 15.567 | .864 | 184638 | .006 |
| South Africa | 14.286 | 20.267 | 103.337 | < .001 | 0 | .866 |
| Spain | 6.667 | 7.172 | 30.005 | < .001 | 1032 | .861 |
| Sweden | 4.651 | 9.152 | 19.025 | < .001 | 58761 | .670 |
| Switzerland | 11.765 | 39.808 | 24.232 | < .001 | 179936 | .311 |
| Taiwan* | 28.571 | 143.120 | 22.277 | < .001 | 1011261 | .415 |
| Turkey | 8.000 | 8.467 | 64.887 | < .001 | 0 | .866 |
| Ukraine | 12.500 | 23.317 | 26.087 | < .001 | 55801 | .389 |
| United Kingdom | 13.636 | 27.280 | 41.193 | < .001 | 65343 | .527 |
| USA | 30.769 | 248.921 | 60.231 | .084 | 472336 | .046 |
| Venezuela | 4.558 | 5.427 | 96.956 | < .001 | 0 | .866 |

Note. For countries marked with *, perceived inequality is larger than objective inequality.

**Table S27.** Underestimation of the Top 10% / Bottom 50% Income Share Ratio in 1999 (Winsorized 90%) (SS1)

| Country | *Median*  Perceived Ratio | *SD*  Perceived  Ratio | Objective  Ratio | *P* | Wilcoxon test Statistic | | Effect size *(r)* |
| --- | --- | --- | --- | --- | --- | --- | --- |
| Australia | 4.000 | 2.057 | 6.586 | < .001 | 141294 | .668 | |
| Austria | 4.000 | 2.750 | 5.459 | < .001 | 111680 | .347 | |
| Bulgaria | 2.000 | 0.748 | 6.098 | < .001 | 0 | .867 | |
| Canada | 4.500 | 2.641 | 8.378 | < .001 | 27005 | .756 | |
| Chile | 5.517 | 6.267 | 17.792 | < .001 | 13318 | .822 | |
| Cyprus | 4.359 | 2.191 | 6.902 | < .001 | 56935 | .667 | |
| Czech Republic | 2.667 | 1.099 | 3.780 | < .001 | 205672 | .593 | |
| France | 3.846 | 1.769 | 5.776 | < .001 | 191358 | .655 | |
| Germany-East | 3.500 | 1.580 | 5.507 | < .001 | 9111 | .702 | |
| Germany-West | 3.750 | 2.124 | 5.507 | < .001 | 58162 | .508 | |
| Hungary | 2.286 | 1.465 | 4.256 | < .001 | 39316 | .683 | |
| Israel | 2.364 | 1.053 | 12.261 | < .001 | 0 | .866 | |
| Japan | 5.000 | 4.065 | 8.490 | < .001 | 44411 | .508 | |
| Latvia | 1.556 | 0.611 | 7.517 | < .001 | 0 | .866 | |
| New Zealand | 4.103 | 2.093 | 7.121 | < .001 | 35857 | .746 | |
| Philippines | 3.611 | 4.753 | 14.026 | < .001 | 8810 | .835 | |
| Poland | 2.143 | 1.297 | 5.108 | < .001 | 4128 | .847 | |
| Portugal | 4.000 | 3.019 | 8.029 | < .001 | 52759 | .678 | |
| Slovakia | 3.333 | 1.474 | 4.328 | < .001 | 141769 | .427 | |
| Slovenia | 2.909 | 1.056 | 5.296 | < .001 | 1981 | .858 | |

**Table S28.** Underestimation of the Top 10% / Bottom 50% Income Share Ratio in 2009 (Winsorized 90%) (SS1)

| Country | *Median*  Perceived Ratio | *SD*  Perceived  Ratio | Objective  Ratio | *P* | Wilcoxon test Statistic | Effect size *(r)* |
| --- | --- | --- | --- | --- | --- | --- |
| Argentina | 2.286 | 1.440 | 12.204 | < .001 | 0 | .866 |
| Australia | 5.000 | 6.281 | 6.360 | < .001 | 388866 | .145 |
| Austria | 4.000 | 3.137 | 6.077 | < .001 | 121454 | .423 |
| Bulgaria | 3.636 | 2.414 | 6.520 | < .001 | 13869 | .668 |
| Chile | 7.273 | 10.138 | 22.209 | < .001 | 114078 | .659 |
| China | 1.667 | 1.004 | 10.651 | < .001 | 0 | .866 |
| Croatia | 3.000 | 0.944 | 7.386 | < .001 | 0 | .866 |
| Cyprus* | 9.412 | 4.223 | 7.352 | < .001 | 387989 | .564 |
| Czech Republic | 3.333 | 1.558 | 3.943 | < .001 | 243646 | .203 |
| Denmark | 3.261 | 1.852 | 4.494 | < .001 | 187732 | .471 |
| Estonia | 2.857 | 1.366 | 7.320 | < .001 | 0 | .866 |
| Finland | 2.632 | 1.194 | 5.944 | < .001 | 1127 | .860 |
| France | 4.000 | 2.940 | 5.668 | < .001 | 856407 | .330 |
| Germany-East | 4.000 | 2.206 | 7.023 | < .001 | 5708 | .736 |
| Germany-West | 3.846 | 2.946 | 7.023 | < .001 | 56297 | .566 |
| Hungary | 2.727 | 1.500 | 5.239 | < .001 | 15682 | .788 |
| Iceland | 3.333 | 1.528 | 4.802 | < .001 | 59477 | .599 |
| Israel | 2.182 | 1.078 | 13.566 | < .001 | 0 | .866 |
| Italy | 3.500 | 1.822 | 5.769 | < .001 | 49698 | .685 |
| Japan | 5.714 | 4.566 | 9.737 | < .001 | 44149 | .541 |
| Korea | 6.667 | 5.987 | 9.909 | < .001 | 325656 | .408 |
| Latvia | 2.400 | 1.349 | 8.009 | < .001 | 0 | .866 |
| Lithuania | 2.500 | 1.283 | 7.888 | < .001 | 0 | .866 |
| New Zealand | 4.000 | 2.171 | 5.857 | < .001 | 82150 | .492 |
| Norway | 2.909 | 1.422 | 4.431 | < .001 | 95822 | .607 |
| Philippines | 4.444 | 5.529 | 13.264 | < .001 | 27132 | .749 |
| Poland | 3.429 | 2.609 | 6.362 | < .001 | 65942 | .581 |
| Portugal | 5.000 | 2.658 | 8.292 | < .001 | 18134 | .705 |
| Russian Federation | 1.667 | 0.718 | 10.854 | < .001 | 0 | .866 |
| Slovakia | 2.501 | 1.322 | 4.530 | < .001 | 36095 | .759 |
| Slovenia | 4.706 | 3.355 | 5.531 | 0.002 | 154755 | .109 |
| South Africa | 10.000 | 12.352 | 25.158 | < .001 | 674536 | .604 |
| Spain | 2.667 | 1.174 | 6.376 | < .001 | 0 | .866 |
| Sweden | 2.250 | 0.664 | 4.730 | < .001 | 0 | .866 |
| Switzerland | 3.333 | 1.866 | 5.165 | < .001 | 98952 | .566 |
| Taiwan* | 7.273 | 5.746 | 6.163 | < .001 | 1209008 | .335 |
| Turkey | 3.636 | 1.976 | 12.358 | < .001 | 0 | .866 |
| Ukraine | 1.500 | 0.464 | 6.566 | < .001 | 0 | .866 |
| United Kingdom | 5.161 | 3.088 | 7.456 | < .001 | 77455 | .472 |
| USA | 6.000 | 5.865 | 10.355 | < .001 | 235872 | .459 |
| Venezuela | 2.143 | 1.075 | 15.760 | < .001 | 0 | .866 |

Note. For countries marked with *, perceived inequality is larger than objective inequality.

**Figure S14.** Objective (red) and perceived (blue) estimates of the top 10% / bottom 50% income inequality in 1999 and 2009 in each country (SS1)
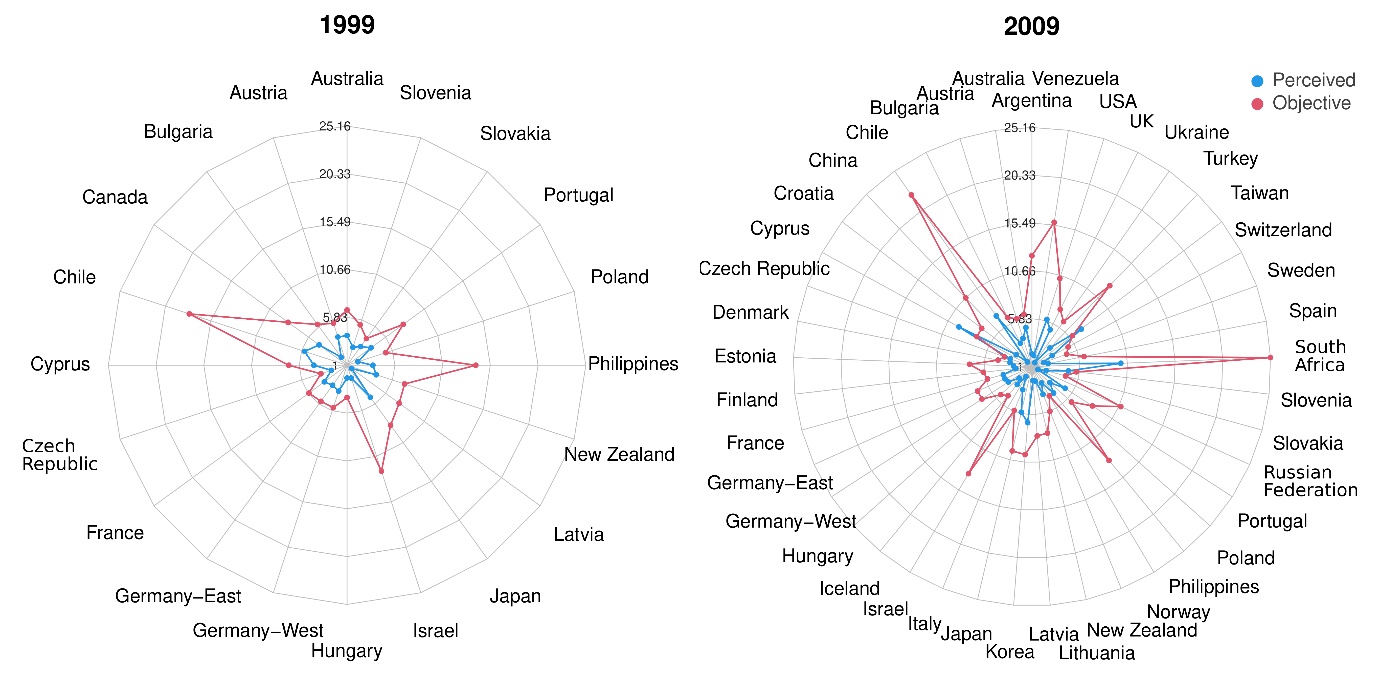


**Table S29.** Underestimation of the Top 1% / Bottom 50% Ratio compared to the underestimation of Top 10% / Bottom 50% Ratio in 1999 (SS1)

| Country | Median  Top 10 misperception | SD  Top 10 misperception | Median  Top 1 misperception | SD  Top 1 misperception | *P* | Wilcoxon test Statistic | Effect Size *(r)* |
| --- | --- | --- | --- | --- | --- | --- | --- |
| Australia | 2.586 | 2.482 | 19.459 | 5.050 | < .001 | 2103547 | .842 |
| Austria | 1.459 | 4.727 | 17.127 | 7.718 | < .001 | 683118 | .754 |
| Bulgaria | 4.098 | 1.064 | 17.941 | 9.475 | < .001 | 511048 | .834 |
| Canada | 3.878 | 4.358 | 28.813 | 71.578 | < .001 | 734066 | .647 |
| Chile | 12.275 | 19.224 | 104.819 | 210.624 | < .001 | 859782 | .738 |
| Cyprus | 2.543 | 3.323 | 20.305 | 3.009 | < .001 | 988438 | .863 |
| Czech Republic | 1.114 | 1.566 | 5.462 | 13.822 | < .001 | 1697068 | .257 |
| France | 1.930 | 3.388 | 9.142 | 25.004 | < .001 | 1995316 | .293 |
| Germany-East | 2.007 | 2.221 | 16.842 | 6.753 | < .001 | 155300 | .747 |
| Germany-West | 1.757 | 2.937 | 16.842 | 6.463 | < .001 | 436026 | .788 |
| Hungary | 1.970 | 2.733 | 2.768 | 32.863 | .016 | 399148 | .058 |
| Israel | 9.897 | 1.822 | 49.125 | 7.042 | < .001 | 1180440 | .866 |
| Japan | 3.490 | 8.598 | 17.064 | 23.473 | < .001 | 344458 | .564 |
| Latvia | 5.962 | 0.900 | 7.107 | 71.582 | .012 | 368475 | .062 |
| New Zealand | 3.019 | 5.063 | 28.000 | 30.668 | < .001 | 942234 | .732 |
| Philippines | 10.415 | 12.587 | 89.664 | 487.198 | < .001 | 948300 | .846 |
| Poland | 2.965 | 3.353 | 10.411 | 54.009 | < .001 | 500163 | .340 |
| Portugal | 4.029 | 7.133 | 22.491 | 15.115 | < .001 | 872927 | .723 |
| Slovakia | 0.995 | 1.561 | 8.237 | 2.382 | < .001 | 1086740 | .845 |
| Slovenia | 2.387 | 2.797 | 7.128 | 13.073 | < .001 | 593242 | .349 |

**Table S30.** Underestimation of the Top 1% / Bottom 50% Income Share compared to the underestimation of Top 10% / Bottom 50% Income Share in 2009 (SS1)

| Country | Median | SD | Median | SD | *P* | Wilcoxon test Statistic | Effect Size *(r)* |
| --- | --- | --- | --- | --- | --- | --- | --- |
|  | Top 10 misperception | Top 10 misperception | Top 1 misperception | Top 1 misperception |  |  |  |
| Argentina | 9.919 | 4.933 | 151.239 | 18.284 | < .001 | 495608 | .861 |
| Australia* | 1.36 | 3247.571 | -3.979 | 271361.374 | < .001 | 841453 | .080 |
| Austria | 2.077 | 4.379 | 13.73 | 47.426 | < .001 | 682017 | .393 |
| Bulgaria | 2.884 | 3.011 | 22.55 | 6.132 | < .001 | 222876 | .835 |
| Chile | 14.936 | 1132.801 | 156.869 | 5293.753 | < .001 | 1727654 | .763 |
| China | 8.985 | 2.857 | 50.229 | 451.478 | < .001 | 1208382 | .735 |
| Croatia | 4.386 | 1.277 | 15.114 | 11.046 | < .001 | 1193247 | .658 |
| Cyprus | -2.06 | 4.711 | 20.176 | 2.641 | < .001 | 939426 | .865 |
| Czech Republic | 0.61 | 2.562 | 12.202 | 12.838 | < .001 | 1136353 | .673 |
| Denmark | 1.233 | 7.924 | 15.672 | 19.639 | < .001 | 1494276 | .725 |
| Estonia | 4.463 | 1.648 | 16.215 | 24.133 | < .001 | 767825 | .700 |
| Finland | 3.312 | 2.331 | 10.818 | 35.443 | < .001 | 474296 | .424 |
| France | 1.668 | 5.644 | 1.445 | 11539.206 | .158 | 2791023 | .021 |
| Germany-East | 3.023 | 3.152 | 18.742 | 492.346 | < .001 | 104129 | .341 |
| Germany-West | 3.177 | 35.937 | 18.742 | 359.285 | < .001 | 435414 | .295 |
| Hungary | 2.511 | 2.523 | 2.638 | 22.056 | .157 | 348842 | .035 |
| Iceland | 1.469 | 2.148 | 7.647 | 9.019 | < .001 | 683312 | .673 |
| Israel | 11.384 | 1.478 | 60.077 | 12.03 | < .001 | 840867 | .845 |
| Italy | 2.269 | 6.063 | 3.185 | 334.399 | < .001 | 509021 | .081 |
| Japan | 4.023 | 17.882 | 23.353 | 61.632 | < .001 | 389918 | .630 |
| Korea* | 3.242 | 18.838 | -4.901 | 41669.621 | .026 | 1160668 | .040 |
| Latvia | 5.609 | 1.934 | 22.019 | 30.535 | < .001 | 564360 | .570 |
| Lithuania | 5.388 | 2.427 | 17.025 | 41.259 | < .001 | 556388 | .485 |
| New Zealand | 1.857 | 3.494 | 15.72 | 29.481 | < .001 | 613096 | .548 |
| Norway | 1.522 | 15.133 | 17.003 | 22.246 | < .001 | 1188949 | .744 |
| Philippines | 8.82 | 1906.301 | 61.304 | 1905.497 | < .001 | 766261 | .811 |
| Poland | 2.933 | 3.966 | 19.725 | 51.719 | < .001 | 643748 | .507 |
| Portugal | 3.292 | 9.832 | 14.112 | 35.852 | < .001 | 279913 | .426 |
| Russian Federation | 9.187 | 0.918 | 53.619 | 138.223 | < .001 | 1046199 | .517 |
| Slovakia | 2.029 | 1.663 | 9.111 | 55.025 | < .001 | 959941 | .566 |
| Slovenia | 0.825 | 4.179 | 0.567 | 10.866 | .012 | 385452 | .061 |
| South Africa | 15.158 | 41.134 | 89.051 | 78.073 | < .001 | 8430245 | .794 |
| Spain | 3.709 | 2.323 | 23.338 | 11.949 | < .001 | 719239 | .759 |
| Sweden | 2.48 | 1.383 | 14.374 | 44.363 | < .001 | 931345 | .675 |
| Switzerland | 1.832 | 4.026 | 12.467 | 269.56 | < .001 | 792855 | .348 |
| Taiwan* | -1.11 | 185.345 | -6.294 | 311.948 | < .001 | 1384936 | .089 |
| Turkey | 8.722 | 3.131 | 56.887 | 15.095 | < .001 | 1581457 | .830 |
| Ukraine | 5.066 | 0.75 | 13.587 | 82.483 | < .001 | 387026 | .368 |
| United Kingdom | 2.295 | 241.701 | 27.557 | 5027.263 | < .001 | 541275 | .526 |
| USA | 4.355 | 273.28 | 29.461 | 1000.327 | < .001 | 1286690 | .249 |
| Venezuela | 13.617 | 2.581 | 92.397 | 14.062 | < .001 | 676472 | .862 |

Note. Countries demarcated with * have underestimation of Top 10% / Bottom 50% ratio larger than Top 1% / Bottom 50% ratio.

**Table S31.** Average income of different occupations across several exemplary countries (SS1)

| Income groups | Cut-off points (World Inequality Database, 2021) | Corresponding occupation used in the paper | Average income of the given occupation | Source |
| --- | --- | --- | --- | --- |
| **USA** |  |  |  |  |
| Top 1% | above $478,644 | Chairman of a large national company (CEO) | $407,430 | [Archived website](https://web.archive.org/web/20230113072256/https:/www.salary.com/research/salary/posting/chairman-salary) |
| Top 10% | above $143,877 | Doctor General Practice | $168,592 | [Archived website](https://web.archive.org/web/20230113072514/https:/www.payscale.com/research/US/Job=Physician_%2F_Doctor%2C_General_Practice/Salary) |
| Bottom 50% | below $44,218 | Shop assistant | $36,290 | [Archived website](https://web.archive.org/web/20230113072856/https:/www.indeed.com/web/20230113072856/https:/www.indeed.com/career/shop-assistant/salaries) |
| Bottom 50% | below $44,218 | Warehouse Worker | $34,371 | [Archived website](https://web.archive.org/web/20230113073214/https:/www.salary.com/research/salary/benchmark/warehouse-worker-i-salary) |
| Bottom 50% | below $44,218 | Farm Worker | $34,854 | [Archived website](https://web.archive.org/web/20230113073351/https:/www.salary.com/research/salary/listing/farm-worker-salary) |
| Bottom 50% | below $44,218 | Dietary Worker | $27,447 | [Archived website](https://web.archive.org/web/20230113073709/https:/www.salary.com/research/salary/listing/dietary-worker-salary) |
| **Germany** |  |  |  |  |
| Top 1% | above €249,405 | Chairman of a large national company (CEO) | €338,111 | [Archived website](https://web.archive.org/web/20230113082143/https:/www.salaryexpert.com/salary/job/chairman-of-the-board-and-ceo/germany) |
| Top 10% | above €75,394 | Doctor General Practice | €84,90€ | [Archived website](https://web.archive.org/web/20230113082355/http:/www.salaryexplorer.com/salary-survey.php?loc=81&loctype=1&job=919&jobtype=3) |
| Bottom 50% | below €30,244 | Shop assistant | €27,000 | [Archived website](https://web.archive.org/web/20230113082532/https:/www.payscale.com/research/DE/Job=Retail_Sales_Assistant/Salary) |
| Bottom 50% | below €30,244 | Warehouse Worker | €33,763 | [Archived website](https://web.archive.org/web/20230113082823/https:/www.erieri.com/salary/job/warehouse-worker/germany) |
| Bottom 50% | below €30,244 | Farm Worker | €27,500 | [Archived website](https://web.archive.org/web/20230113083042/http:/www.salaryexplorer.com/salary-survey.php?loc=81&loctype=1&job=29&jobtype=1) |
| Bottom 50% | below €30,244 | Dietary Worker | €33,400 | [Archived website](https://web.archive.org/save/http:/www.salaryexplorer.com/salary-survey.php?loc=81&loctype=1&job=403&jobtype=3) |
| **Hungary** |  |  |  |  |
| Top 1% | above 25,089,248 Ft | Chairman of a large national company (CEO) | 50,342,551 Ft | [Archived website](https://web.archive.org/web/20230118183728/https:/www.salaryexpert.com/salary/job/chairman-of-the-board-and-ceo/hungary) |
| Top 10% | above 7,301,058 Ft | Doctor General Practice | 13,900,000 Ft | [Archived website](https://web.archive.org/web/20230113141652/http:/www.salaryexplorer.com/salary-survey.php?loc=98&loctype=1&job=13&jobtype=2) |
| Bottom 50% | below 4,015,828 Ft | Shop assistant | 2,700,000 Ft | [Archived website](https://web.archive.org/web/20230113141945/http:/www.salaryexplorer.com/salary-survey.php?loc=98&loctype=1&job=672&jobtype=3) |
| Bottom 50% | below 4,015,828 Ft | Warehouse Worker | 3,544,448 Ft | [Archived website](https://web.archive.org/web/20230120125319/https:/www.salaryexpert.com/salary/job/warehouse-worker/hungary) |
| Bottom 50% | below 4,015,828 Ft | Farm Worker | 3,560,000 Ft | [Archived website](https://web.archive.org/web/20230118184458/http:/www.salaryexplorer.com/salary-survey.php?loc=98&loctype=1&job=29&jobtype=1) |
| Bottom 50% | below 4,015,828 Ft | Dietary Worker | 4,045,462 Ft | [Archived website](https://web.archive.org/web/20230118184835/https:/www.salaryexpert.com/salary/job/dietary-assistant/hungary) |
| **Australia** |  |  |  |  |
| Top 1% | above AU$402,124 | Chairman of a large national company (CEO) | AU$415,743 | [Archived website](https://web.archive.org/web/20230118183728/https:/www.salaryexpert.com/salary/job/chairman-of-the-board-and-ceo/hungary) |
| Top 10% | above AU$171,313 | Doctor General Practice | AU$306,718 | [Archived website](https://web.archive.org/web/20230118182942/https:/www.salaryexpert.com/salary/job/general-practitioner/australia) |
| Bottom 50% | below AU$62,233 | Shop assistant | AU$51,044 | [Archived website](https://web.archive.org/web/20230119084535/https:/www.salaryexpert.com/salary/job/shop-assistant/australia) |
| Bottom 50% | below AU$62,233 | Warehouse Worker | AU$45,370 | [Archived website](https://web.archive.org/web/20230118181501/https:/www.salaryexpert.com/salary/job/warehouse-assistant/australia/sydney) |
| Bottom 50% | below AU$62,233 | Farm Worker | AU$42,246 | [Archived website](https://web.archive.org/web/20230118181346/https:/www.salaryexpert.com/salary/job/farm-worker/australia) |
| Bottom 50% | below AU$62,233 | Dietary Worker | AU$43,929 | [Archived website](https://web.archive.org/web/20230118180657/https:/www.salaryexpert.com/salary/job/food-service-dietary-aide/australia/sydney) |
| **Argentina** |  |  |  |  |
| Top 1% | above ARS 8,715,282 | Chairman of a large national company (CEO) | ARS 17,820,997 | [Archived website](https://web.archive.org/web/20230118180340/https:/www.salaryexpert.com/salary/job/chairman-of-the-board-and-ceo/argentina) |
| Top 10% | above ARS 2,234,556 | Doctor General Practice | ARS 6,156,677 | [Archived website](https://web.archive.org/web/20230119085035/https:/www.salaryexpert.com/salary/job/doctor/argentina) |
| Bottom 50% | below ARS 638,608 | Shop assistant | ARS 261,000 | [Archived website](https://web.archive.org/web/20230118175501/http:/www.salaryexplorer.com/salary-survey.php?loc=10&loctype=1&job=672&jobtype=3) |
| Bottom 50% | below ARS 638,608 | Warehouse Worker | ARS 280,000 | [Archived website](https://web.archive.org/web/20230118175243/https:/www.salary.com/research/ar-salary/benchmark/warehouse-worker-salary/ar) |
| Bottom 50% | below ARS 638,608 | Farm Worker | ARS 326,000 | [Archived website](https://web.archive.org/web/20230118174650/http:/www.salaryexplorer.com/salary-survey.php?loc=10&loctype=1&job=29&jobtype=1) |
| Bottom 50% | below ARS 638,608 | Dietary Worker | ARS 361,000 | [Archived website](https://web.archive.org/web/20230118174313/http:/www.salaryexplorer.com/salary-survey.php?loc=10&loctype=1&job=403&jobtype=3) |
| **Korea** |  |  |  |  |
| Top 1% | above ₩147,695,824 | Chairman of a large national company (CEO) | ₩251,373,408 | [Archived website](https://web.archive.org/web/20230119085401/https:/www.salaryexpert.com/salary/job/chairman-of-the-board-and-ceo/south-korea) |
| Top 10% | above ₩55,234,424 | Doctor General Practice | ₩119,225,985 | [Archived website](https://web.archive.org/web/20230118172628/https:/www.salaryexpert.com/salary/job/doctor/south-korea) |
| Bottom 50% | below ₩24,032,422 | Shop assistant | ₩25,116,490 | [Archived website](https://web.archive.org/web/20230118172334/https:/www.salaryexpert.com/salary/job/shop-assistant/south-korea) |
| Bottom 50% | below ₩24,032,422 | Warehouse Worker | ₩14,800,000 | [Archived website](https://web.archive.org/web/20230122134233/http:/www.salaryexplorer.com/salary-survey.php?loc=114&loctype=1&job=11425&jobtype=3) |
| Bottom 50% | below ₩24,032,422 | Farm Worker | ₩20,009,584 | [Archived website](https://web.archive.org/web/20230118165344/https:/www.salaryexpert.com/salary/job/farm-worker/south-korea) |
| Bottom 50% | below ₩24,032,422 | Dietary Worker | ₩24,747,762 | [Archived website](https://web.archive.org/web/20230118165227/https:/www.salaryexpert.com/salary/job/food-service-worker/south-korea/seoul) |

*Supplementary Analysis in General Discussion*

We explored whether changes in the actual concentration of incomes held by the top 1% between 1999 and 2009—the years participants were surveyed in the ISSP dataset—were related to support for redistribution.

Objective income inequality was calculated the same way as detailed above in SS1, using data obtained from the World Inequality Database and ISSP. The redistribution measure was taken from the International Social Survey Program. Respondents answered the question “Do you think people with high incomes should pay larger share of their income in taxes?” on a five-point Likert scale (1 = much larger share, 5 = much smaller share) both in 1999 and 2009. To increase clarity, we have reverse coded the items in a way that higher values represent higher preference for redistribution.

We examined whether changes in the actual concentration of incomes held by the top 1% between 1999 and 2009—the years participants were surveyed in the ISSP dataset, in the countries where we had data available for both years—were related to support for redistribution, but did not find a statistically significant effect (*b* = .002, *se* = .004, *CI95%* = [-.006, .010], *p* = .643).
